# Challenges in reducing maternal and neonatal mortality in Niger: an in-depth case study

**DOI:** 10.1136/bmjgh-2023-011732

**Published:** 2024-05-06

**Authors:** Almamy Malick Kante, Lamou Ousseini Youssoufa, Aida Mounkaila, Yahaha Mahamadou, Assanatou Bamogo, Safia S Jiwani, Elizabeth Hazel, Abdoulaye Maïga, Melinda Kay Munos, Shelley Walton, Yvonne Tam, Neff Walker, Nadia Akseer, Heather Jue Wong, Mohamed Moussa, Abdoua Elhadji Dagobi, Nasreen S Jessani, Agbessi Amouzou

**Affiliations:** 1 Johns Hopkins University Bloomberg School of Public Health, Baltimore, Maryland, USA; 2 Institut National de la Statistique, Niamey, Niger; 3 Direction des Statistiques Sanitaires, Ministère de la Santé Publique, Niamey, Niger; 4 Université Abdou Moumouni de Niamey, Niamey, Niger; 5 Knowledge, Impact and Policy Unit, Institute of Development Studies, Brighton, UK

**Keywords:** Maternal health, Child health, Health policies and all other topics, Health systems, Public Health

## Abstract

**Introduction:**

Recent modelled estimates suggest that Niger made progress in maternal mortality since 2000. However, neonatal mortality has not declined since 2012 and maternal mortality estimates were based on limited data. We researched the drivers of progress and challenges.

**Methods:**

We reviewed two decades of health policies, analysed mortality trends from United Nations data and six national household surveys between 1998 and 2021 and assessed coverage and inequalities of maternal and newborn health indicators. Quality of care was evaluated from health facility surveys in 2015 and 2019 and emergency obstetric assessments in 2011 and 2017. We determined the impact of intervention coverage on maternal and neonatal lives saved between 2000 and 2020. We interviewed 31 key informants to understand the factors underpinning policy implementation.

**Results:**

Empirical maternal mortality ratio declined from 709 to 520 per 100 000 live births during 2000–2011, while neonatal mortality rate declined from 46 to 23 per 1000 live births during 2000–2012 then increased to 43 in 2018. Inequalities in neonatal mortality were reduced across socioeconomic and demographic strata. Key maternal and newborn health indicators improved over 2000–2012, except for caesarean sections, although the overall levels were low. Interventions delivered during childbirth saved most maternal and newborn lives. Progress came from health centre expansion, emergency care and the 2006 fee exemptions policy. During the past decade, challenges included expansion of emergency care, continued high fertility, security issues, financing and health workforce. Social determinants saw minimal change.

**Conclusions:**

Niger reduced maternal and neonatal mortality during 2000–2012, but progress has stalled. Further reductions require strategies targeting comprehensive care, referrals, quality of care, fertility reduction, social determinants and improved security nationwide.

**WHAT IS ALREADY KNOWN ON THIS TOPIC**

- Despite a challenging physical, social, political and economic environment, Niger significantly reduced mortality among children under 5 since 2000 through the expansion of community case management of childhood illnesses, campaigns for the distribution of insecticide-treated bednets and vitamin A and expansion of nutrition rehabilitation centres.
- Gains in maternal and neonatal mortality were modest during the same period and their drivers and challenges have not been researched.

**WHAT THIS STUDY ADDS**

- Maternal and neonatal mortality declined slightly during 2000–2012, driven by a broad strategy of expansion of access to healthcare for women and children through the construction of primary integrated health centres and community health posts, the 2006 fee exemptions policy for mothers and children and access to basic emergency obstetric and newborn care, resulting in increased coverage of maternal and newborn health services.
- Since 2012, progress in most coverage indicators stalled and inequalities persisted with an increase in neonatal mortality, particularly in the most disadvantaged groups.
- Access to emergency obstetric and neonatal care remained poor; insufficient and unequal distribution of health workforce and infrastructure, high fertility and decade-long insecurity appear to impede longer term progress in maternal and neonatal mortality.

**HOW THIS STUDY MIGHT AFFECT RESEARCH, PRACTICE OR POLICY**

- Further decline in maternal and neonatal mortality will require Niger to prioritise targeted strategies that increase access to comprehensive emergency obstetric and newborn care; improve referral and quality of care; significantly control high fertility; pay attention to social determinants such as women’s education and empowerment; and address the security concerns.
- In-depth research is needed to understand barriers to access to Emergency Obstetric and Newborn Care, expansion of human resources for health, insecurity and environmental impact.

## Introduction

Niger is a landlocked West African country, lying on the southern edge of the Sahara Desert with 80% of its land occupied by the desert.^1 2^ With a population of 23.3 million in 2019, almost doubled since 2000,^3^ Niger is one of the poorest countries in the world, consistently ranked as last on the Human Development Index,^4^ with about half of its population living under the poverty line.^5^ Despite these challenges, Niger successfully reduced under-5 mortality rate (U5MR).^6 7^ A study found a 5.1% annual rate of reduction (ARR) in U5MR between 1998 and 2009. This progress was mainly driven by reductions in postneonatal deaths.^8^,^10^ Gains in neonatal survival were modest. Newborn health intervention coverage during antenatal care (ANC), childbirth and postnatal care (PNC) increased slightly but was still low. An analysis of verbal and social autopsy data found that about two-thirds of neonates died from infection while 20% died of intrapartum birth complications.^11^ Neonatal deaths were associated with poor-quality ANC and PNC; high prevalence of maternal complications, including pregnancy and labour and delivery complications linked to early neonatal deaths, with poor care seeking for these complications. Thus, while Niger has been successful at rolling out strategies to prevent child deaths, major gaps existed in the expansion of care for mothers and newborns.

Recent estimates by the United Nations Inter-agency Group for Child Mortality Estimation (UN-IGME) estimated a slow neonatal mortality decline with only a 22% reduction between 2000 and 2010 and a stall at 34 per 1000 live births since 2010.^12^ More progress may have occurred for maternal mortality based on the UN estimates, which estimated a 50% decline in maternal mortality ratio (MMR) from 867 to 441 per 100 000 live births between 2000 and 2020. However, these estimates were based on extrapolated trends between 2000 and 2010 that relied on only two available data points. Although the UN estimates suggest a deceleration in the reduction of under-5 mortality, Niger faces major challenges in reducing largely its maternal and neonatal mortality.^13 14^ This paper reviews progress in maternal and neonatal mortality in Niger since 2000 and investigates drivers and policy challenges that explain the slow progress observed.

Niger witnessed peaks and troughs in government leadership which affected the prioritisation of health system. In 2000, the country issued a declaration for rural development ensuring access to basic services, including maternal and newborn health (MNH). A subsequent regime established in 2006 a fee exemptions policy for all pregnant women and all children under 5 and the expansion of community health posts in rural and remote areas. However, since the early 2010s, the emergence of terrorist groups in the Sahel poses significant security challenges, particularly in Diffa, Maradi, Tahoua and Tillaberi, that led to changes in government priorities.^1^ The health system in Niger is predominantly public and organised in a decentralised three-tier hierarchical structure (district or primary, intermediate or regional, and central or national levels) (online supplemental appendix figure 1). It prioritises the expansion of access to primary healthcare (PHC) and referral. Maternal and neonatal care are integrated at all levels of service delivery. To increase access to healthcare at the community level, the health system has been organised further from the district level to integrated health centres (IHCs), which in turn supervise the activities of the health posts to which community health workers are linked.^1^

## Methods

### Data sources

We used data from six national household surveys: three Demographic and Health Surveys (DHS 1998, 2006 and 2012)^15^,^17^ and three government household surveys (*Enque Santé Mortalité,* ESM, 2010, *Etude Nationale d’Evaluation d’Indicateurs Socio-economques et Demographiques*, ENISED, 2015 and *Enquete Nationale sur la Fécondité et la Mortalité des Enfants de Moins de Cinq Ans, ENAFEME,* 2021)^18^,^20^; and two Service Availability and Readiness Assessment (SARA 2015 and 2019) surveys.^13 14^ We accessed maternal and neonatal mortality modelled estimates from the UN^12 21^ for national trends assessment (online supplemental appendix table 1).

We conducted an extensive review of country health reports, statistical yearbooks, national health accounts and global databases (World Bank, WHO and UN Population Division) to identify data on health system programmes, financing, human resources for health (HRH) and other contextual factors. These were complemented with a literature search in PubMed, Scopus and country government websites to capture the literature on programmes and policies implemented since 2000. We also exploited findings from the 2011 and 2017 Emergency Obstetric and Newborn Care (EmONC) assessment reports.^14 22^ Actual data were not available for reanalysis.

Between August and November 2021, we carried out 31 key informant interviews (KII) with health programme managers from the Ministry of Public Health (MoPH) and other national and international institutions who were involved in policies and programme implementation in the past 20 years in Niger.

### Analysis

We computed the ARR in MMR between 2000 and 2020 and in neonatal mortality rate (NMR) between 2000 and 2021 using an exponential growth formula with a constant negative rate of reduction. We relied on measured empirical mortality from available household surveys for disaggregated and equity analysis. For NMR, we pooled birth history modules from the six national household surveys, restricted to births in the past 15 years preceding each survey. We assessed the level of consistency in NMR across the surveys prior to pooling the data. The pooled data included a total of 152 876 births. We computed NMR on 3-year periods at the national level and 5-year periods at the subnational level (region and place of residence) and for characteristics that were unlikely to change over time for each birth (eg, maternal education, place of birth, birth risk factors, household wealth quintile). The birth risk composition distinguished births with no risk, unavoidable risk and single or multiple risks (online supplemental appendix table 2).

We assessed trends separately for 2000–2012 and 2013–2021. The year 2012 was used as the mid-way cut-off period because of the DHS 2012. The analysis used the sampling weights to account for the sampling design.^23^ We computed SEs around the mortality estimates using jackknife methods.^24^ Maternal mortality estimates were extracted from the country survey reports.^16 17 19^

We assessed changes in the coverage of MNH indicators and their disaggregation by same stratifiers as for mortality using the reanalysed database from the International Center for Equity in Health.^25^ Absolute equity gaps, annual percentage point (pp) changes and equity patterns were visualised using equiplots.

To assess changes in the quality of care, we computed facility ANC and delivery readiness using SARA 2015 and SARA 2019. The ANC readiness was computed as an arithmetic average of the availability of 22 essential items across five domains for ANC.^26^ We calculated the facility readiness score for delivery services from 20 basic emergency obstetric and newborn care (BEmONC) items across three domains. We also generated a comprehensive emergency obstetric and newborn care (CEmONC) readiness score covering 19 items, restricted to facilities providing caesarean section (C-section) and blood transfusion services (online supplemental appendix table 3).

We ecologically linked available health facility surveys with household surveys to assess readiness-adjusted coverage of interventions offered during ANC and childbirth. We then linked a woman’s report of a facility delivery to the average labour and delivery readiness score of that facility type in the woman’s region of residence to compute readiness-adjusted delivery care estimates (online supplemental appendix 1).

We assessed the contribution of health interventions to maternal and neonatal survival by conducting complementary analyses. First, we assessed the role of fertility changes on maternal and newborn lives saved and mortality decline between 2000 and 2017 using the Jain decomposition method.^27^ We then analysed the contribution of intervention coverage to changes in maternal and neonatal mortality using the *Lives Saved Tool* (LiST).^10^

Finally, the qualitative interviews were transcribed and coded using NVivo and organised by the main health system and emerging major themes, and along categories of drivers of a conceptual framework.^28^ Data from the KII were also triangulated with data gathered through desk and literature review (online supplemental appendix 1).

### Patient and public involvement

Patients or the public were not involved in the design, conduct, reporting or dissemination plans of this research. However, the Niger MoPH and the National Statistics Office were involved in this research. The findings were shared with the Ministry of Health (MoH) and stakeholders.

## Results

### Mortality levels, trends and inequalities

**Figure 1:**
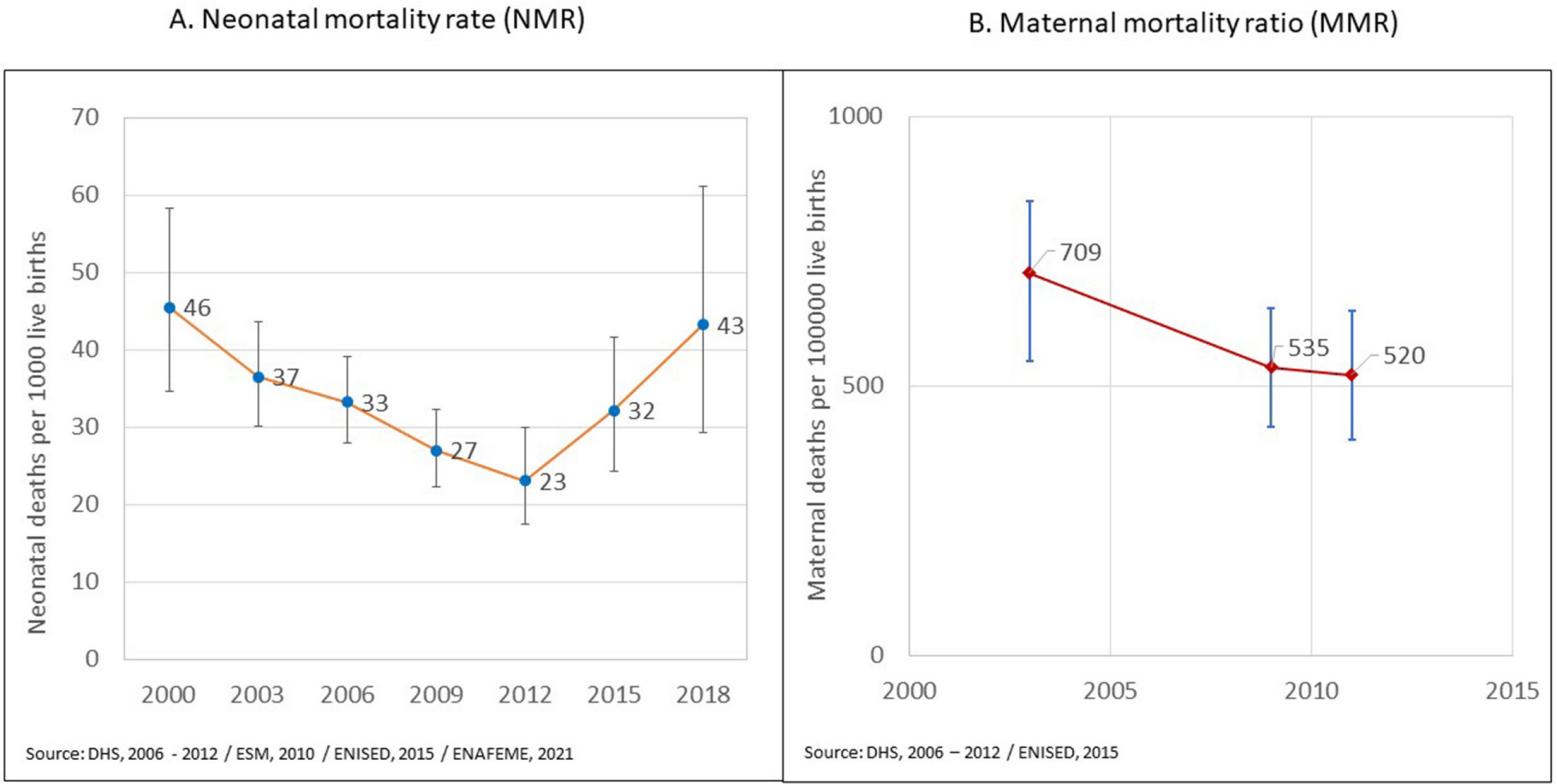
Trends in neonatal mortality rate (NMR) (A) and maternal (pregnancy-related) mortality ratio (MMR) (B) in Niger, estimates from national household surveys. DHS, Demographic and Health Survey; ENISED, Etude Nationale d’Evaluation d’Indicateurs Socioeconomiques et Demographiques.

Based on surveys, Niger reduced its NMR from 46 per 1000 (95% CI 35–58) to 23 (95% CI 17–30) between 2000 and 2012. However, NMR has increased since 2012, reaching 43 (95% CI 29–61) in 2018 (figure 1). The 2022 UN-IGME models estimated NMR at 44 (95% CI 39–50) in 2000, declining to 34 (95% CI 30–39) in 2010 and stagnating until 2021, suggesting only mere 24% decline between 2000 and 2010 (online supplemental appendix figure 2).

The survey-based MMR declined from 709 per 100 000 (95% CI 576–872) during 2000–2006 to 535 (95% CI 425–645) during 2007–2012, then stalled to 520 (95% CI 400–639) during 2009–2015 (figure 1). The UN-Maternal Mortality Estimation Interagency Group estimated MMR at 867 (95% CI 704–1074) in 2000, declining to 441 (95% CI 305–655) in 2020, almost 50% decline or 3.4% ARR. This decline was based on the extrapolation of trends during 2000–2010, which relied only on two data points (online supplemental appendix figure 3).

**Figure 2:**
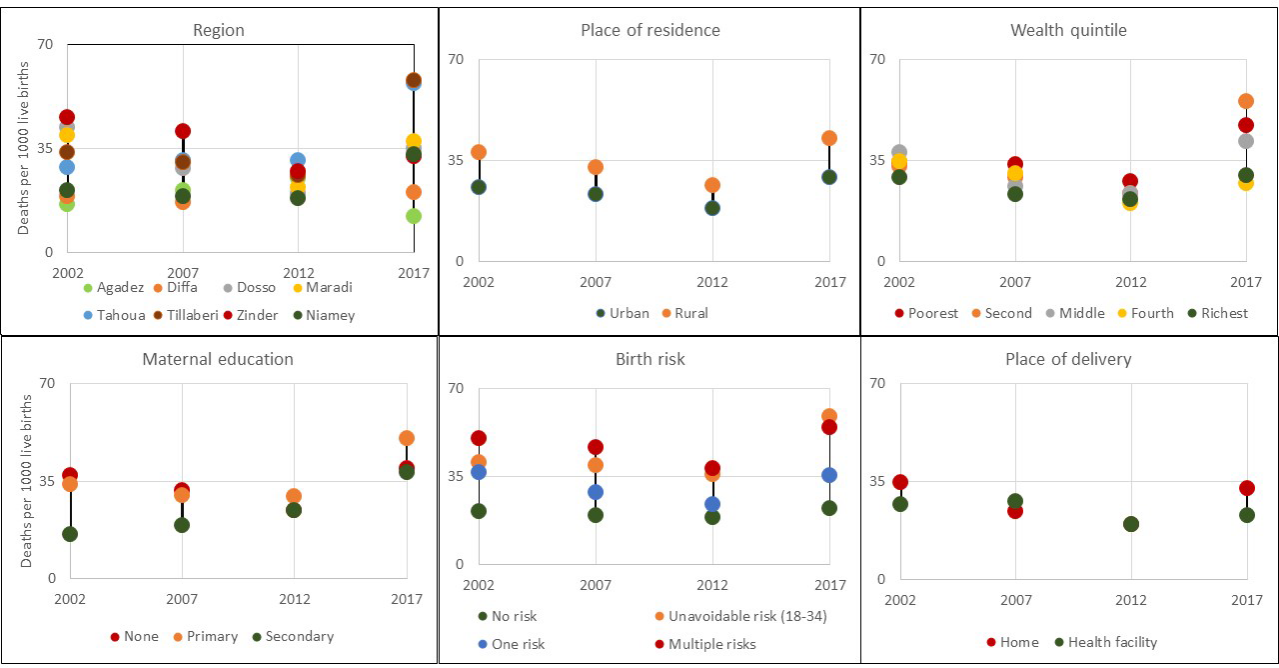
Inequality trends in neonatal mortality rate in Niger, 2000–2017 (5-year period).

Based on surveys, inequality in NMR reduced across all analysed strata of the population. The gap by region reduced substantially from 42 points in 2000 (between Niamey and Zinder) to 11 points in 2012 (between Diffa and Tahoua), although the top and bottom regions differed for each period. The gap by place of residence (urban vs rural) halved from 22 to 10 points between 2000 and 2012. The gap by wealth quintiles was also reduced. However, the pattern was irregular in 2000 such that the poorest was not the group with the highest NMR. The gap by maternal education almost closed from 30 to 5 points gap between mothers with no education and those with secondary or more education between 2000 and 2012. The gap by birth risk reduced slightly with no-risk births registering the lowest NMR while multiple-risk births were the highest. The gap by place of delivery reduced over time and by 2012, NMR was similar between home and facility delivery (figure 2).

During the past decade, NMR gaps widened in almost all strata reversing back to differences observed in 2000. Between 2012 and 2017, the gap increased substantially to 46 points between Agadez and Tillaberi or Tahoua, 13 points between urban and rural areas, 17 points between wealthiest and poorest households, 32 points between no-risk births and multiple-risk births and 10 points between health facility and home delivery.

### Coverage of MNH interventions

**Figure 3:**
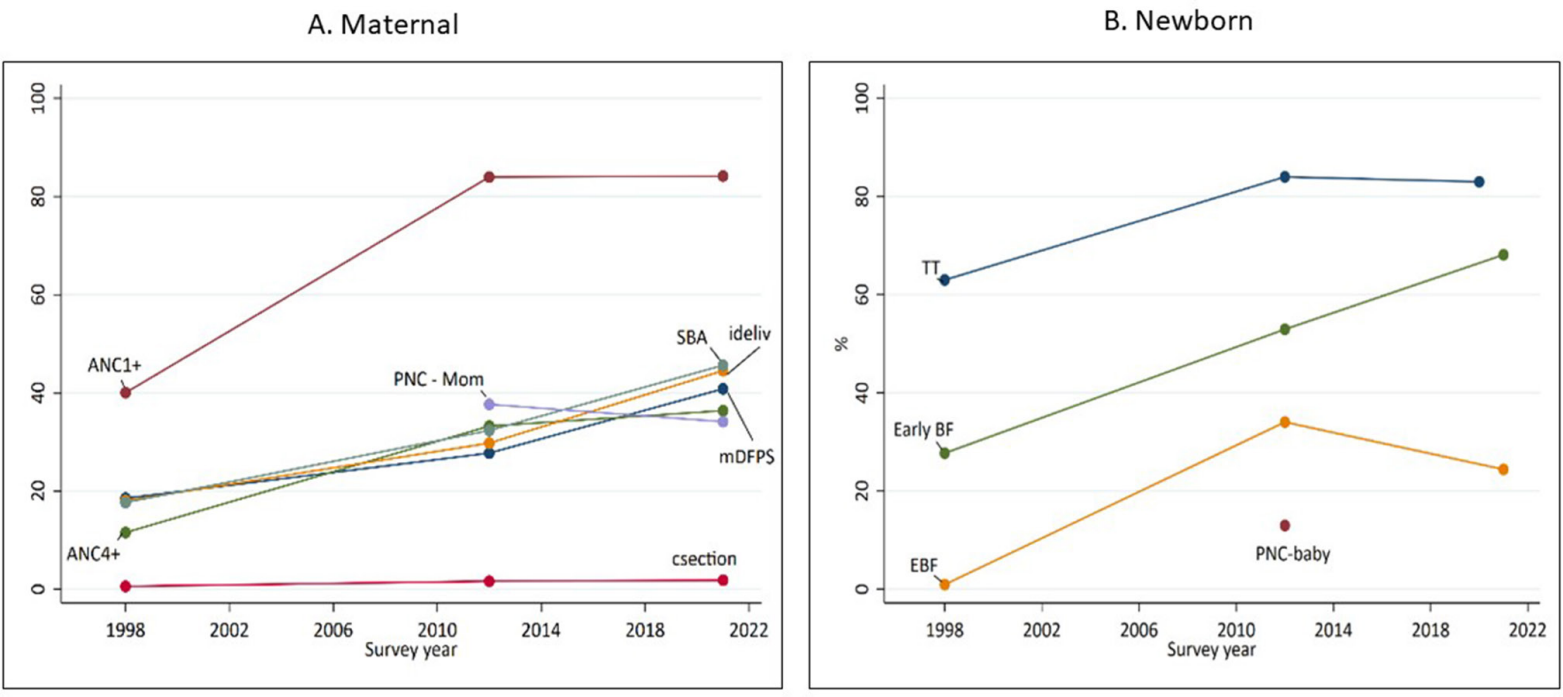
Coverage trends in maternal (A) and newborn (B) health (MNH) indicators; Demographic and Health Survey (DHS) 1998, 2012 and ENAFEME 2021 surveys. mDFPS: Demand for family planning satisfied by modern contraceptive methods (modem methods indude pills, condoms (male and female), intrauterine deviœe, sterilization (male andfemale), injectables, implant, diaphragm, spermicidal agents, patch and emergency contraception); ANC1+: One or more visits of antenatal care; ANC4+: Four or more visits of antenatal care; ideliv: Birthoccurred at a health institution/health facility; PNC-mom: women received a postnatal check-up within two days post-delivery; SBA: skilled birth attendant; csection: œsarean section; EBF: Infants less thanone month of age reœived only breastmilk in the previous 24 hours; TT: baby was born protected from tetanus toxoid infection; Early BF: Baby was breastfed in the first hour after delivery; PNC-baby: Babyreceived a postnatal check-up within two days post-delivery

Niger experienced substantial increases in the coverage of MNH indicators in 2000–2012. The demand for family planning satisfied with a modern contraceptive method doubled from 19% in 1998 to 41% in 2021 (figure 3). The coverage of ANC visits increased substantially between 1998 and 2012 (ANC1: from 40% to 84%, ANC4: from 12% to 33%). It remained unchanged since 2012 (figure 3).

Health facility delivery rates increased from 18% in 1998 to 30% in 2012 and 45% in 2021. Skilled birth attendant rates increased from 18% in 1998 to 29% in 2012 and 39% in 2021. In 1998, 83% of facility births occurred in government hospitals. However, this proportion decreased over time and by 2021 most facility births, 70% occurred in IHCs (online supplemental appendix figure 4). The use of C-sections remained poor (below 2%) since 1998. PNC for women and babies is suboptimal and only 39% of women received care within 2 days of delivery in 2012 which remained stagnant (figure 3).

More babies were born protected from tetanus infection (from 63% in 1998 to 83% in 2012, stagnating until 2021). More mothers are initiating breast feeding early (from 28% in 1998 to 53% in 2012 and 68% in 2021). Exclusive breast feeding for neonates has improved since 1998, from 1% to 34% in 2012 but dropped to 24% in 2021 (figure 3).

Socioeconomic inequity in coverage has not improved overall, except for ANC1 for which the gap almost closed by 2021. The wealth health gap is the widest and most persistent for institutional delivery and skilled attendants at birth, setting the poorest and the wealthiest apart by over 60 pp. Indicators with very low coverage such as C-section, PNC for newborns and exclusive breast feeding had no or small wealth inequity gap. Women residing in urban areas or those with a secondary or higher education had higher coverage than others. Considerable differences were found with most coverage indicators by region. However, the inequity gaps have not reduced over time across regions, maternal education and place of residence, except for ANC1 (online supplemental appendix figures 5–8).

### Availability and quality of maternal and newborn care

EmONC assessments revealed that Niger has very low coverage of BEmONC service, although an increase was observed between 2010 and 2017.^22 29^ Based on recommended norms of five BEmONC facilities for 500 000 people, of which one must be a CEmONC facility, Niger had only 0.10 BEmONC and 1 CEmONC facilities per 500 000 people in 2010. The BEmONC coverage rate increased to 0.97 in 2017, while the CEmONC coverage decreased to 0.85. Major disparities exist between regions and between places of residence. In 2017, only 33% of EmONC facilities were in rural areas and none offered CEmONC functions. While IHCs cover most deliveries, only 29% of them offered BEmONC services in 2017. In rural areas, 3% of deliveries occurred in a BEmONC facility. Services such as newborn resuscitation, vacuum-assisted deliveries and removal of products are critically deficient. Challenges included the availability of competent human resources, equipment and drugs.^22 29^

Data from SARA in 2019 indicated that 82% of facilities offered ANC services and 78% provided labour and delivery services. IHCs account for over 90% of facilities offering ANC and delivery services (online supplemental appendix figure 9). Quality of care, based on facility readiness, remained unchanged between 2015 and 2019. The national-level average facility readiness score remained at 70% for ANC, 63% for normal deliveries (BEmONC) and 75% for comprehensive obstetric care among CEmONC-designated facilities (online supplemental appendix table 4). Regional trend variations were minimal for ANC readiness, but for BEmONC readiness, the largest improvements were observed in the Diffa region (58–70% between 2015 and 2019), whereas Tahoua experienced the largest decline in readiness (62–52%) (online supplemental appendix figures 10 and 11).

Due to lack of early data, we cannot assess changes since 2000. However, self-reported content of ANC suggested an overall improvement in the mean ANC content score by 25 pp between 2006 and 2021. Counselling for pregnancy complications during ANC visits substantially increased from 24% to 57% between 2006 and 2012 (online supplemental appendix figure 12).

All women accessing delivery care have not however received optimal quality care. Ecological linking between household survey and health facility data suggested a 21 pp readiness gap between readiness-adjusted delivery care (38%) and service contact for institutional deliveries (59%) in 2015, and a 19 pp gap in 2021, with large variations across regions, Diffa with the lowest pp gap and Niamey the highest (online supplemental appendix figures 13 and 14).

### Contribution of health intervention coverage to changes in maternal and neonatal mortality

Crude birth rates in Niger declined from 54 to 46 per 1000 between 2000 and 2019, according to the UN estimates.^3^ Our Jain’s decomposition analysis revealed that changes in birth rates contributed to only 15% of maternal lives saved. Changes in birth risk composition contributed to only 9% (online supplemental appendix figures 15 and 16). Thus, the large part of maternal and newborn lives saved and the decline in MMR and NMR were due to health programme factors other than fertility.

**Figure 4:**
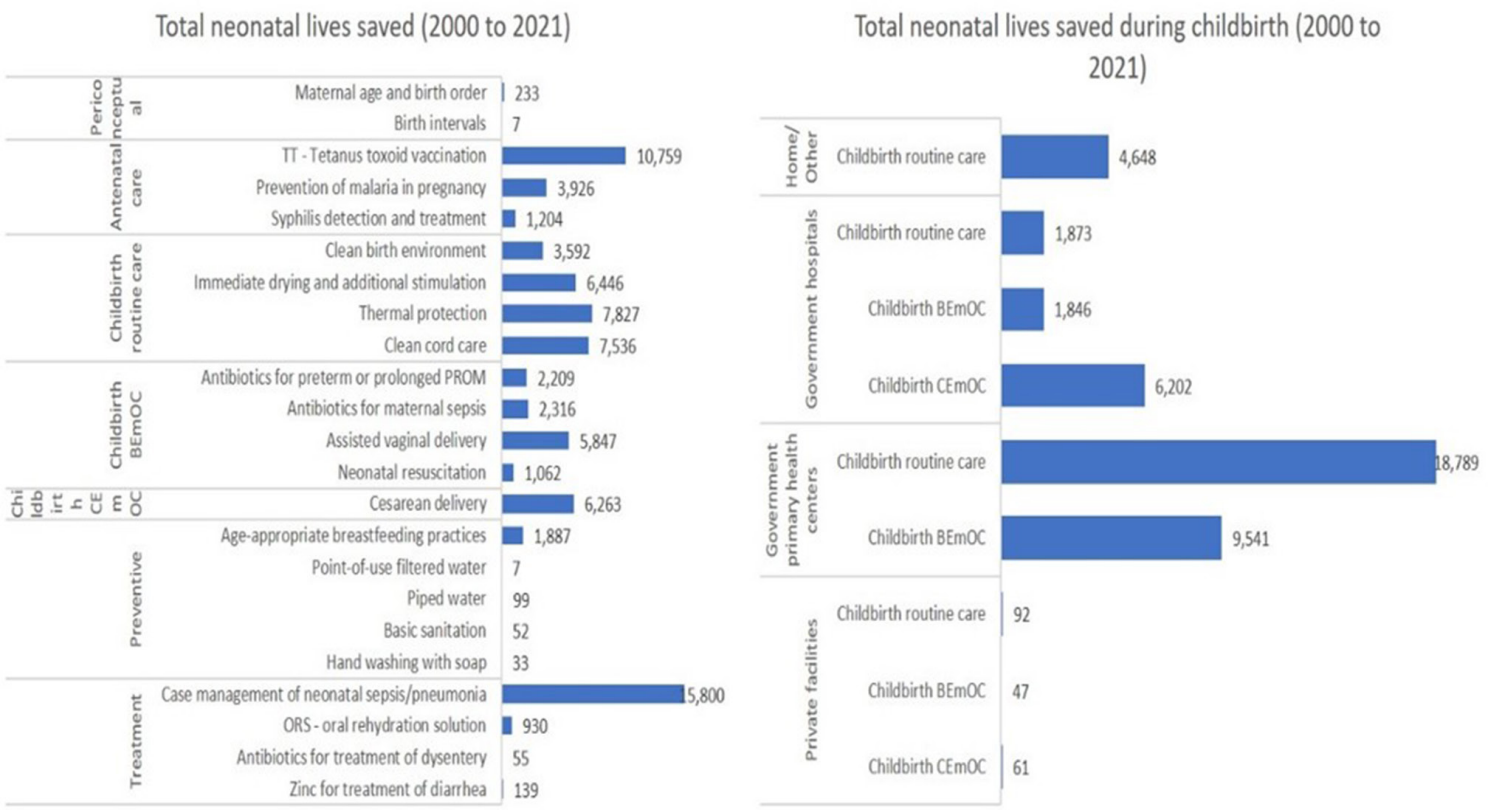
Neonatal lives saved by interventions, groups of interventions by the continuum of care and facility types. Estimations obtained using the Lives Saved Tool (LiST) model. BEmOC, basic emergency obstetric care; CEmOC, comprehensive emergency obstetric care. PROM, Premature rupture of membranes.

The LiST model estimated that a total of 78 230 neonatal lives were saved between 2000 and 2021, of which 43 100 (55%) were from interventions during childbirth (figure 4). Most neonatal lives saved were due to routine care-specific interventions during childbirth. The interventions that saved most neonatal lives were case management of neonatal sepsis/pneumonia, followed by tetanus toxoid vaccination. LiST mortality projections were consistent with the UN-IGME’s estimated mortality reduction between 2000 and 2020, suggesting that the estimated mortality changes were primarily due to changes in intervention coverage (online supplemental appendix figure 17).

**Figure 5:**
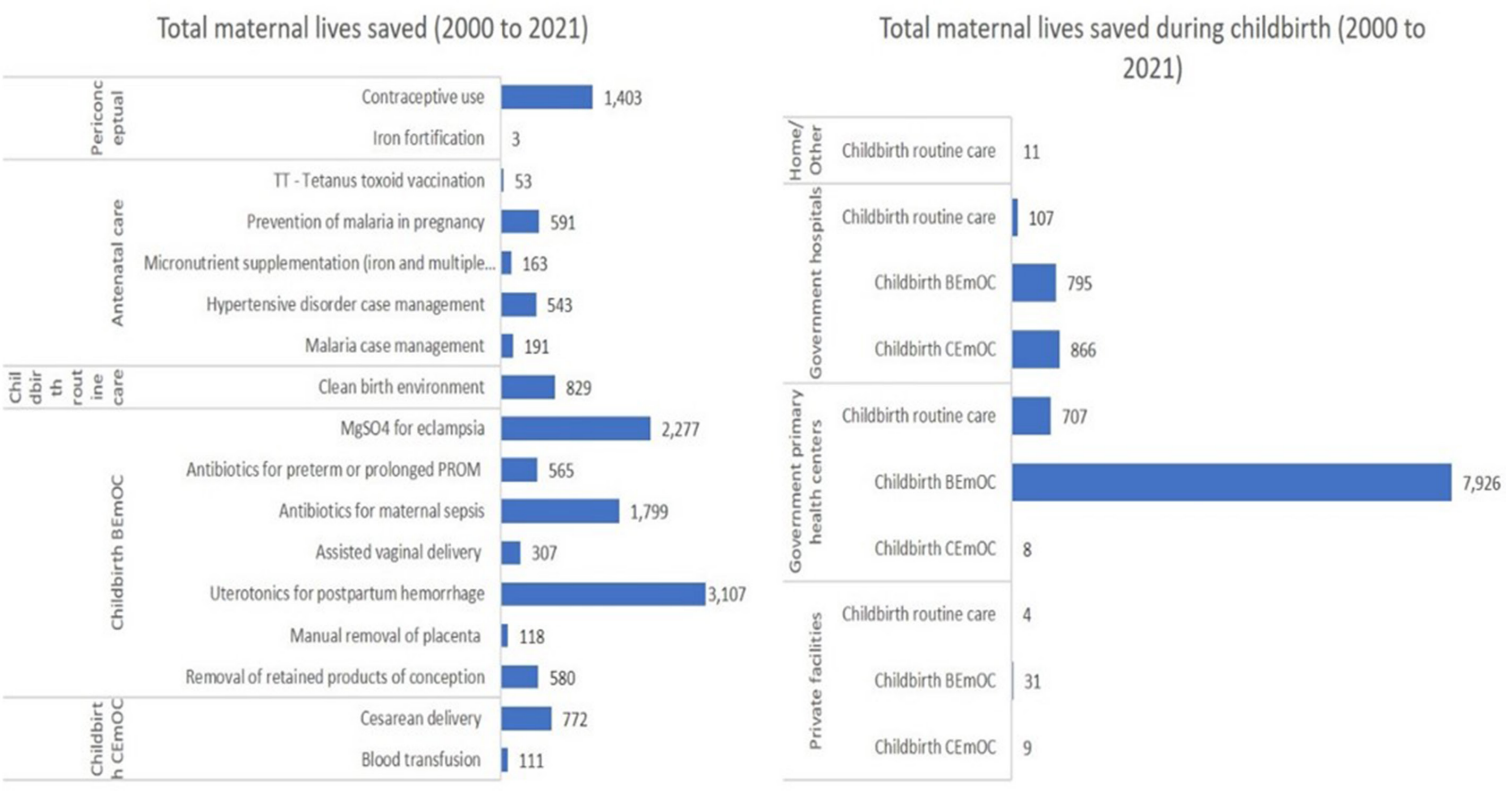
Maternal lives saved by interventions, groups of interventions by the continuum of care and facility types. Estimations obtained using the Lives Saved Tool (LiST) model. BEmOC, basic emergency obstetric care; CEmOC, comprehensive emergency obstetric care. PROM, Premature rupture of membranes.

The LiST model estimated that a total of 13 410 maternal lives were saved between 2000 and 2021, of which 10 465 (78%) were from interventions during childbirth (figure 5). Most maternal lives saved were due to BEmONC-specific interventions during childbirth. The interventions that led to most maternal lives saved were uterotonics for postpartum haemorrhage, followed by magnesium sulfate for eclampsia, using antibiotics to treat maternal sepsis, and contraceptive use. Most maternal lives saved during childbirth were at IHC (online supplemental appendix figure 18).

### Drivers of neonatal mortality change

**Figure 6:**
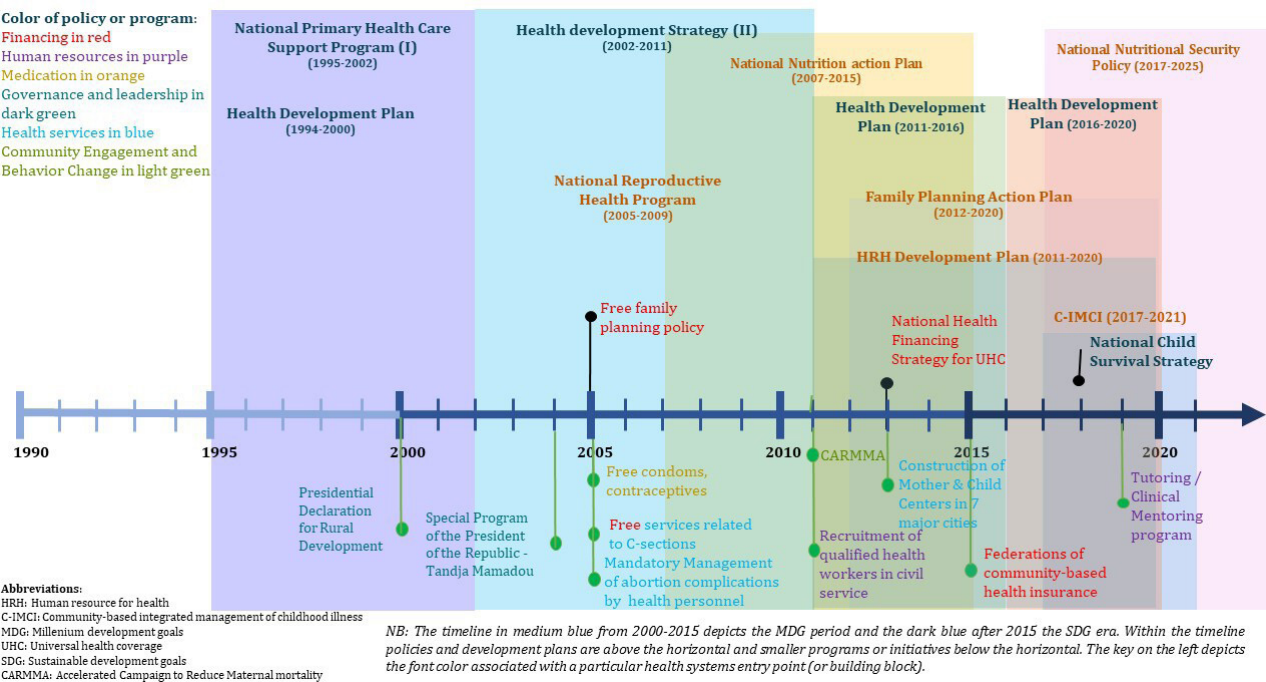
Key maternal and newborn policies and programmes timeline in Niger. C-IMCI, community-based integrated management of childhood illness; C-section, caesarean section; HRH, human resources for health; MDG, Millennium Development Goals; SDG, Sustainable Development Goals; UHC, universal health coverage. CARMMA, Accelerated Campaign to Reduce Maternal Mortality

Numerous policies in Niger contributed to the population’s health, including maternal and child health. The timeline in figure 6 suggests a concerted effort since the 1990s, accelerated after 2005, which seems to be the beginning of a period that led to several high-level strategies and action plans—each of which overlapped with other strategies, policies and programmes that likely together contributed to improvements of maternal and child health. The government prioritised the expansion and strengthening of PHC such as IHCs, subdivided into IHC-I and IHC-II linked to an expanded network of health posts and community health workers.^1^ Between 2000 and 2012, Niger doubled the number of IHCs from 400 to 856 and reached 1194 by 2020, of which 869 were IHC-I and 325 were IHC-II. The number of functional health posts expanded to 2451 in 2012 before reducing to 2320 in 2020.^30 31^ Mobile clinics were introduced for the nomadic population. In 2013, maternal and child referral health centres were constructed in all regions to address high-risk pregnancies and EmONC.

Niger adopted the National Health Financing Strategy in 2012.^30^ Four sectors drove the health financing: the public sector financing through the government, household out-of-pocket (OOP) payments, community-based health insurance schemes and private health insurance (online supplemental appendix figure 19). Despite the increase in allocation to the health sector, the government has struggled to reach the 15% allocation of the national budget to the health sector recommended by the Abuja declaration and the 10% recommended by WHO.^31^ In addition, efforts were not sustained in the past decade with the health expenditure falling by half and then stalling since 2011.^1^ This is illustrated by the quote below from an MoH respondent.

We participated in the evaluation of health policies. It was found that there was only one year in which Niger injected 10% of its budget into health financing. For the rest, the rates of budget allocation to the health sector have varied between 5% and 9%. So, we can see that there is still a lot to be done to reach this 15%. (Director, MoH1)

Private insurance is minimal, with a 3% penetration, accounting only for 0.3% of health spending.^30^ OOP constitutes the largest proportion of health financing (online supplemental appendix figure 19, left). Changes in OOP spending and health spending per capita are depicted in online supplemental appendix figure 19. User fee exemptions implemented for women and children in 2006 to specific priority diseases and to address priority concerns^32^ alleviated the burden related to the cost of care.^33^ However, this policy faced many challenges; only 60% of invoices between 2007 and 2019 have been reimbursed.^34^

Niger falls short of the WHO recommended standards for HRH.^35^ The ratios by category of staff are among the lowest in the world (online supplemental appendix figure 20). Efforts to improve the availability of HRH have been successful, with an increased number of medical doctors and nurses/midwives around 2011 due to the presidential initiative to recruit highly qualified healthcare workers to support the PHC system deployment.^9^ However, these efforts have not been sustained in the recent decade. The equitable distribution of healthcare workers remained a challenge due to implementation issues that did not account for the environmental, personal and social conditions of healthcare personnel, particularly in rural areas.^36^

The insecurity context since the 2010s in the Sahel may have partially hampered progress in Niger due to the government’s change in priorities and also the closure or destruction of health facilities and schools in affected regions which may have impacted the access to MNH services.^1^ An MoPH programme director and a physician put it in this way:

Insecurity has been the priority of Niger’s governments for several years in terms of efforts and funding. This means that sectors as important as health have been somewhat neglected and no longer receive the funding they deserve. Insecurity has therefore given the State a different dynamic in terms of funding priorities, and it is obliged to redirect certain resources. As a result, when we go to the health centers, it is no longer like before; the free services that were well established in many centers no longer exist. (Director, MoPH2)

Recently, one of the major constraints in the health sector has been insecurity. Today, health services in many of the so-called red zones of our country are no longer functional. They are closed or destroyed, and in some cases, it is the agents who are forced to leave their posts. So, women and children in these areas no longer have access to health care. In the areas where displaced persons are staying, the available health structures and services do not fully cover the enormous needs of the displaced and the host populations. Thus, we can say that maternal and neonatal health is taking a serious hit due to insecurity, and the reduction of maternal and neonatal mortality in the areas concerned is encountering enormous difficulties. (Physician 1)

## Discussion

Niger reduced maternal and neonatal mortality over 2000–2012 due to major policy and programme efforts. The UN model-based estimates of NMR declined by 22% over 2000–2010 and stagnated since then. However, available empirical surveys suggest an increase in the past decade.^19 20^ MMR is estimated to continue declining, but the trend is based on extrapolation of the decline observed in 2000–2010. The coverage of MNH interventions improved following a vigorous policy implementation and community-based child survival programme between 2000 and 2012, which in turn significantly reduced the NMR. However, the coverage of MNH interventions remained relatively low since 2012 and most indicators have not changed in the past decade.

Empirical surveys suggest that Niger succeeded in gradually closing the NMR inequality gaps across social strata between 2000 and 2012.^15^,^18^ Gaps by regions, place of residence, wealth quintiles and maternal education have substantially narrowed between 1998 and 2012. This progress occurred through faster improvement in the most disadvantaged groups such as the poor, the rural or the least educated populations, indicating success in reaching these groups with MNH programmes and obtaining higher returns in terms of health benefits. While MNH coverage levels remained comparatively lower in these groups, their increase yielded higher survival gains than well-off groups.

Progress has been achieved mainly by leveraging key health pillars, notably in increasing access to health facilities and prioritising the expansion of PHC. Galvanised by a presidential declaration in 2000, priority was given to expanding access to care at the community level through the expansion of health posts linked to IHC and to BEmONC. The number of basic emergency obstetric care facilities increased rapidly. The number of IHCs doubled between 2000 and 2012. The 2006 fee exemptions policy for pregnant women and children under 5 was pivotal in increasing access to reproductive, maternal, newborn and child health services for vulnerable populations. However, the change in OOP was small. There were challenges with reimbursing health facilities, and patients having to subsidise costs of their care regardless.^3237^,^39^

Our LiST analysis showed that interventions delivered during childbirth saved most maternal and newborn lives. Case management of neonatal sepsis/pneumonia, tetanus protection at birth and neonatal resuscitation contributed to the most newborn lives saved. Reducing postpartum haemorrhage, eclampsia and maternal sepsis contributed to the most maternal lives saved.

Progress observed in the 2000 decade contrasted with stagnation and some reversals in the past decade. Niger’s health system strategy faced numerous challenges that appeared to be reversing the hard-won gains in maternal and neonatal mortality. The number of new IHCs has slightly increased over the past decade, but the NMR has not declined proportionally. The shortage of HRH and their equitable distribution have remained a challenge during that period. In addition, the expansion of health facilities was fraught with challenges such as ‘limited technical platform for essential medical care and the necessary incentives to maintain doctors in rural areas’.^36^ In 2014, of the 280 doctors recruited, only 180 were in their posts, including 119 in Niamey.^40^ Many initiatives to overcome the limitation of healthcare staff failed because of poor implementation, mainly not accounting for environmental, personal and social conditions of healthcare personnel, particularly in rural areas.^36^ Therefore, the ratio of medical doctors has even decreased by half and the ratio of nurse/midwives has stalled since 2012. This has affected the facility readiness for ANC, normal deliveries (BEmONC) and CEmONC during the recent decade. Furthermore, priority to access to basic primary care was not matched with similar priority to CEmONC and no increase in access to C-section deliveries was observed. The CEmONC coverage decreased and all available CEmONC facilities were in urban settings.^14^ In addition, deliveries in hospitals have been redirected over time to lower level health facilities, particularly the IHCs, where readiness and quality of care were suboptimal, and referrals faced substantial obstacles. The national-level average facility readiness score has not improved for ANC, normal deliveries (BEmONC) and comprehensive obstetric care (CEmONC).

The persistent high fertility rates have not benefited the decline in maternal and neonatal mortality and appeared to be counteracting the hard-earned gains. Niger’s total fertility rate is the highest in the world, estimated at 7.7 children per woman in 2000, and declined only slightly to 7.3 in 2012 and 6.7 in 2020.^3^ Adolescents’ fertility rate (aged 15–19) remained high around 200 children per 1000 since 2000.^41^ The annual number of births has nearly doubled between 2000 and 2020, adding over a million births annually. Niger’s total population doubled in size in 20 years.^3 41^ Our fertility decomposition analysis showed that the modest decline in crude birth rates between 2000 and 2017 contributed to 15% of maternal and neonatal lives saved, while changes in birth risk composition contributed to only 10% of lives saved. These changes are modest compared with the number of annual pregnancies and births in the population.

Progress in social determinants of health was mixed over the past three decades. Poverty has reduced from 79% to 41%.^1^ The gross domestic product per capita (current US$) increased by 29% from 1990 to 2021, after a major drop in 2000.^42^ Urbanisation has remained stagnant at 16% since 1990.^43^ The proportion of women aged 15+ with no schooling remained very high (80% in 2020) and only reduced from 89% since 1992, while the proportion of women with secondary or more education increased from 3% to 8.5% over the same period.^44^ Religious and cultural beliefs and traditions have long shaped age at marriage, reproductive health, family planning choices and birthing decisions. Approximately 89% of young girls marry before they reach the age of 18, and about a third of adolescent girls 15–19 years old have given birth or have been pregnant for the first time.^45 46^ In 2019, the gender inequality index for Niger was 0.64, ranked 154 out of 162, one of the poorest countries for women’s equality worldwide.^4^ Indicators for women’s empowerment have remained steady with only minor improvements in the past two decades. Early marriage and multiple poorly spaced pregnancies put a woman at higher risk for negative birth outcomes and mortality for the mother and newborn.^45 47^

The past decade has also seen a rise in insecurity related to the protracted sporadic terrorist attacks in the Sahel that destabilised Niger’s priorities. The government has revised its national budget allocation with an increase in the security budget by 2%.^1^ Studies in the Sahel showed that terrorist attacks significantly affect the access to maternal healthcare services.^48^,^50^ However, we did not observe a substantial decline in MNH coverage and mortality in the affected regions (Diffa, Maradi, Tahoua and Tillaberi).^1^ NMR in the recent period has increased in almost all regions, except Agadez and Diffa. More research is needed to better quantify and understand the impact of terrorist attacks on MNH intervention and mortality.

The findings discussed in this paper bear some limitations, primarily based on data availability challenges. There were limited neonatal mortality data in the recent period to confirm the increase in NMR since 2012. The National Institute of Statistics implemented national mortality surveys in 2015 and 2021 but did not receive the same technical assistance as the previous DHS.^19 20^ There may therefore exist differential implementation quality across the surveys.^51^ *The DHS Program* has not validated the 2017 Niger DHS data due to data quality issues.^52^ The 2021 household survey was conducted with support from *Utica International*. Data quality issues have not been reported but the recent increase in neonatal mortality has raised many questions. The relationship between insecurity and maternal healthcare access and its effect on maternal and neonatal mortality has not been fully examined in Niger. Data on maternal mortality came from three household surveys and are based on sibling histories. There were no maternal mortality data in recent periods. There is only one 2010 survey that collected child causes of death using verbal autopsy.^11^ Finally, health system data, especially HRH, were severely limited and inconsistent.^28^

## Conclusion

Niger’s success in reducing maternal and neonatal mortality in 2000–2012 was driven by the prioritisation of policies and programmes to expand access to care for women and children, and efforts to withstand the counteracting negative effects of a vulnerable environment. The 2000 presidential declaration for rural development and universal healthcare, followed by the 2006 fee exemptions policy for maternal and child health services, accelerated actions that resulted in the construction and strengthening of IHCs and health posts. Integration of BEmONC interventions into IHCs also sought to enhance maternal and child health. The decentralisation of healthcare to the community level focused on rural areas where the need was greatest and investment to improve access to care have contributed to reaching the poor. While the strategy has generated positive impacts in 2000–2012 for maternal and newborn survival, the survival gains have not been sustained in the subsequent decade, calling for further interrogation of the current PHC strategy in a context of continued high fertility, speedy population growth and the unstable political, security and climate environment.

## Supplementary material

10.1136/bmjgh-2023-011732 online supplemental file 1

## Data Availability

Data are available upon reasonable request. Data may be obtained from a third party and are not publicly available.
