# Supplementary material for: Challenges in reducing maternal and neonatal mortality in Niger: an in-depth case study
Source: BMJ Glob Health. 2024 May 6;9(Suppl 2):e011732. doi: 10.1136/bmjgh-2023-011732 (PMC11085984; doi:10.1136/bmjgh-2023-011732)
Supplement: online supplemental file 1 [file bmjgh-2023-011732supp001.pdf]

Appendix 1.

Quantitative data analysis

We computed the annual rate of reduction (ARR) in MMR between 2000 and 2017 and in NMR between 2000 and 2019 using an exponential growth formula with a constant negative rate of reduction. The trends in modeled estimates were used for comparison across countries in West Africa. We relied on measured empirical mortality for analysis. For NMR, we pooled birth history modules from the six national household surveys, restricted to births in the past ten years preceding each survey. We assessed the consistency of estimates of NMR across the surveys prior to pooling the data. The pooled data included a total of 152 876 births. We computed 95% confidence intervals (CIs) using the Jackknife non-parametric method. We computed NMR on three-year periods at national and five-year period at subnational levels (region and place of residence) and for characteristics that were unlikely to change over time for each birth (e.g., maternal education, place of birth, birth-risk factors, household wealth quintile).We analyzed inequalities in NMR by the same characteristics. The birth risk composition distinguished births with no risk, with unavoidable risk, and single or multiple risks (see definition in Appendix Table 2).

We assessed trends separately for 2000-2012 and 2013-2021. The year 2012 was used as the midway cutoff period because of the 2012 DHS. The analysis used the sampling weights to account for the sampling design.[22] We computed standard errors around the mortality estimates using Jackknife non-parametric methods.[23] Maternal mortality estimates were extracted from the country survey reports. Niger does not have empirical data on causes of neonatal and maternal deaths, besides a follow-up to the 2010 survey that collected child causes of death using verbal autopsy.[24]

We assessed changes in the coverage of standard MNH indicators and their disaggregation by similar stratifiers as for mortality (except for birth risks) using the reanalyzed database from the International Center for Equity in Health (ICEH).[25] Absolute equity gaps, annual percentage points (pp) changes, and equity patterns were visualized using equiplots.

To assess changes in the quality of care, we computed facility ANC and delivery readiness using SARA 2015 and SARA 2019. The ANC readiness was computed as an arithmetic average of the availability of 22 essential items across five domains for ANC [26]: equipment, diagnostics, medicines and commodities, basic amenities, and human resources (Appendix Table 3).

We calculated the facility readiness score for delivery services from 20 basic emergency obstetric and newborn care (BEmONC) items across three domains: equipment and supplies, medicines and commodities, human resources and guidelines. We also generated a Comprehensive emergency obstetric and newborn care (CEmONC) readiness score covering 19 items, restricted to facilities providing C- section and blood transfusion services (Appendix Table 3).

We ecologically linked available health facility surveys with household surveys to assess readiness-adjusted coverage measures of interventions offered during antenatal care and childbirth that are not available in household surveys alone. The linking was possible only for delivery services for which place of delivery information was collected in the household surveys. Ecological linking was done by facility type and geographic region: first, we matched the facility types reported in the household survey to the corresponding facilities in the health facility survey. We then linked a woman's report of a facility delivery to the average

labor and delivery readiness score of that facility type in the woman's region of residence, to compute readiness-adjusted delivery care estimates. For ANC, where linking was not feasible, we generated an ANC content score from six content interventions: urine and blood tests, counseling for pregnancy complications, blood pressure measurement, iron/folic acid supplementation, and intermittent preventive treatment of malaria (IPTp). Self-reported content of ANC was measured as the proportion of women with at least one ANC contact during their last pregnancy who reported receiving these key content interventions as part of ANC. We calculated an overall score out of 6 for the receipt of these 6 ANC content items, with one point assigned to each of the 6 content items, and the score was converted into a percentage.

We assessed the contribution of distal, intermediate, and proximate factors on maternal and neonatal mortality by conducting three sets of complementary analyses. First, we assessed the role of fertility changes on maternal and newborn lives saved and mortality decline between 2000 and 2017 using the proposed decomposition method by Jain.[27] Fertility changes are key factors of change in maternal and neonatal mortality. Fertility decline implies changes in birth rates and in birth risk composition. Because both maternal mortality ratio and neonatal mortality rates are expressed per birth unit, decline in birth rates does not necessarily imply a decline in the risk of death. Thus, a decline in birth rates will only affect the number of maternal lives saved. However, when birth risk composition changes toward lower risk births, it can induce a decline in MMR and NMR.

We then analyzed the contribution of intervention coverage to changes in maternal and neonatal mortality using the lives saved tool (LiST),[10] a mathematical modeling tool that uses changes in coverage intervention coverage to estimate the number of lives saved. LiST also decomposes the contribution of specific interventions to the total lives saved.

Qualitative data analysis

A total of the 31 key informant interviews conducted between August 9 and November 29, 2021, and 25 could be transcribed and the other six were subject to written summaries.

Coding of responses

The first task was to create general categories corresponding to the major themes contained in the interview guide. Each category was then subdivided into sub-categories or sub-themes, which we then added to as we processed the data. Each sub-theme is subdivided in turn, according to the desired level of detail. This results in sub-categories that are finer and more numerous than the initial categories from which they were derived, and that express increasingly precise aspects of the survey. This process of splitting up the interviews allows us to put the data in order and thus prepare for the data analysis work.

Data analysis

The analysis was carried out in two stages. The first stage grouped the data according to their affinity and thus classified the points of view into "families", distinguishing between those that converge and those that diverge on the different questions of the survey. The second step consisted in triangulating the data by considering these points of view in relation to each other. This cross-reading of the data made it possible, depending on the case, to confirm, qualify or refute the points expressed by one or another interviewee.

The triangulation was not limited to the data collected in the survey. In fact, the information obtained from the literature search allowed us to better understand the general problem of maternal and neonatal health

and, consequently, to take a step back from the positions defended by our interlocutors. In other words, the literature search provided a reading grid that considerably facilitated the analysis of the data.

Litterature review

The literature review focused on the evolution of the health system policy and program development and implementation in Niger between 2000 and 2020 to address several themes including the organization of care, infrastructure, human resources, financing, drugs, governance, etc. In most cases, these documents were the subject of reading notes that made it possible to identify the content of the policies and programs discussed, the context in which they were formulated, the mechanisms for their implementation, their results and their implications for maternal and neonatal health (MNH). The literature review thus allowed us to develop a general description of the organization of care for the period 2000-2020 and to develop a detailed chronology of MNH health policies in Niger for the same period. Certain themes, such as health financing and family planning, were the subject of in-depth analyses. These analyses are included in the appendix of this study.

Appendix 2.

Acronyms

ADB	African Development Bank
AfDB-Health II	African Development Fund's Health Care Improvement Project
ANC	Antenatal care
ARI	Acute respiratory infection
ARR	Annual rate of reduction
BF	Breastfeeding
BMI	Body mass index
CC	Community clinic
CNERS	Comite National d'Ethique pour la Recherche en Sante
CNSS	National Social Security Fund
CSI	Integrated Health Centers
CSME	Public maternal and child centers
DH	District Hospitals
DHS	Demographic Health Surveys
ENISED	Etude Nationale d'Evaluation d'Indicateurs Socioeconomiques et Demographiques
GDP	Gross domestic product
GII	Gender inequality index
GNI	Gross national income
HDP	Health Development Plan
HRDP	Human Resources Development Plan
HW	Health workers
HRH	Human resources for health
ICEH	International Center for Equity in Health
IGME	Interagency Group for Child Mortality Estimation
IHC	Integrated Health Centers
IHME	Institute for Health Metrics and Evaluation
LiST	Lives Saved Tool
MCEE	Maternal Child Epidemiology Estimation Group
MCHC	Maternal and child health centers
MICS	Multiple Indicator Cluster Surveys
MMEIG	Maternal Mortality Interagency Group
MMR	Maternal mortality ratio
MNH	Maternal and newborn health
MOF	Ministry of Finance
MOPH	Ministry of Public Health
NGO	Nongovernmental organization
NHFS	National health financing strategy
NMR	Neonatal mortality rate
OOP	Out-of-pocket (expenditures)
PAIPCE	Private Initiative and Employment Creation Support Program
PHC	Primary healthcare
PP	Percentage points
PNC	Postnatal care
SARA	Service Availability and Readiness Assessment
SBA	Skilled birth attendant

SPA	Service Provisional Assessment
TFR	Total fertility rate
UN	United Nations
UNFPA	United Nations Population Fund
USD ($)	United States dollars
VAT	Value added tax
WHO	World Health Organization

Appendix Figure

Appendix Figure 1: The administrative and service delivery components of the tiered health system in Niger Organization, 2020

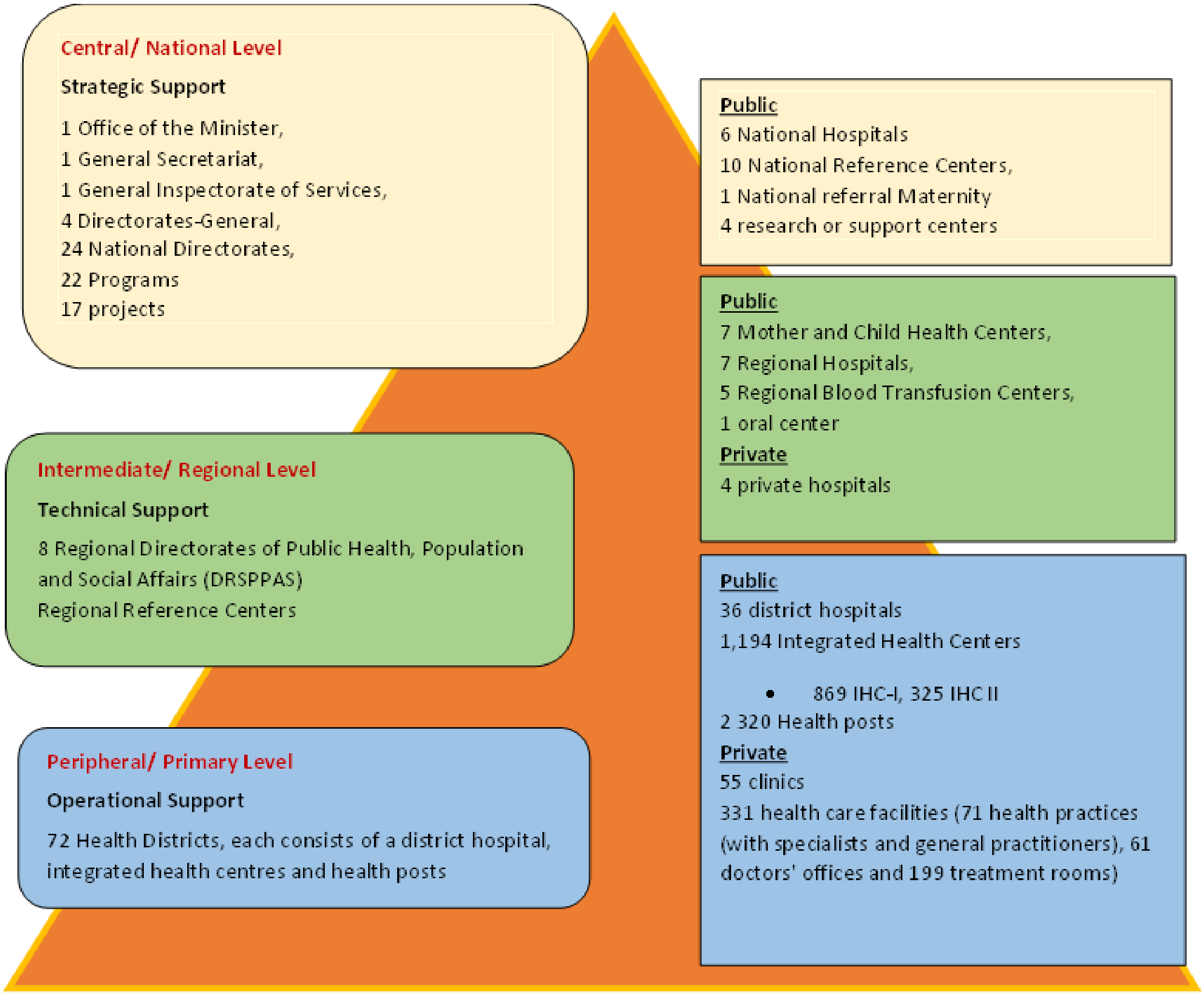

Appendix Figure 2: Trends in neonatal mortality rates in Niger, Estimates from UN-IGME, 2020

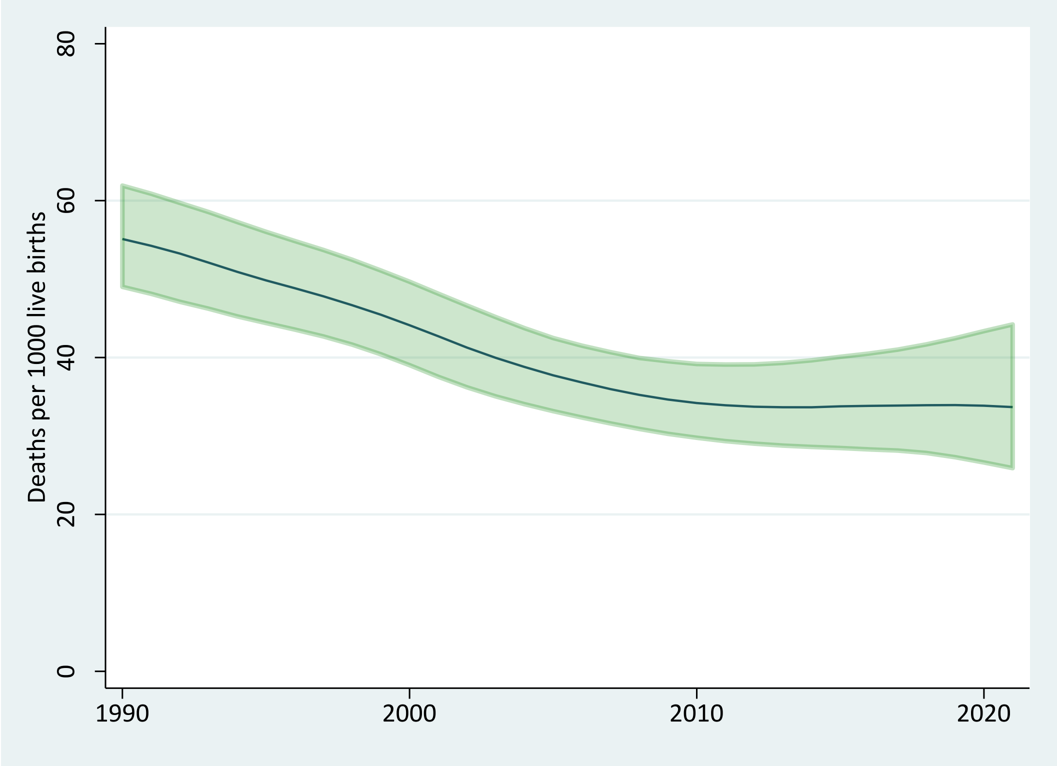

Niger made only modest progress in declining the neonatal mortality rate, from 44 deaths per 1000 live births in 2000 to 34 in 2021, an annual rate reduction (ARR) of 1.3, according to the United Nations – Inter agency Mortality Group (UN-IGME) estimates.

Appendix Figure 3: Trends in maternal mortality ratio in Niger, estimates from UN-MMEIG, 2020

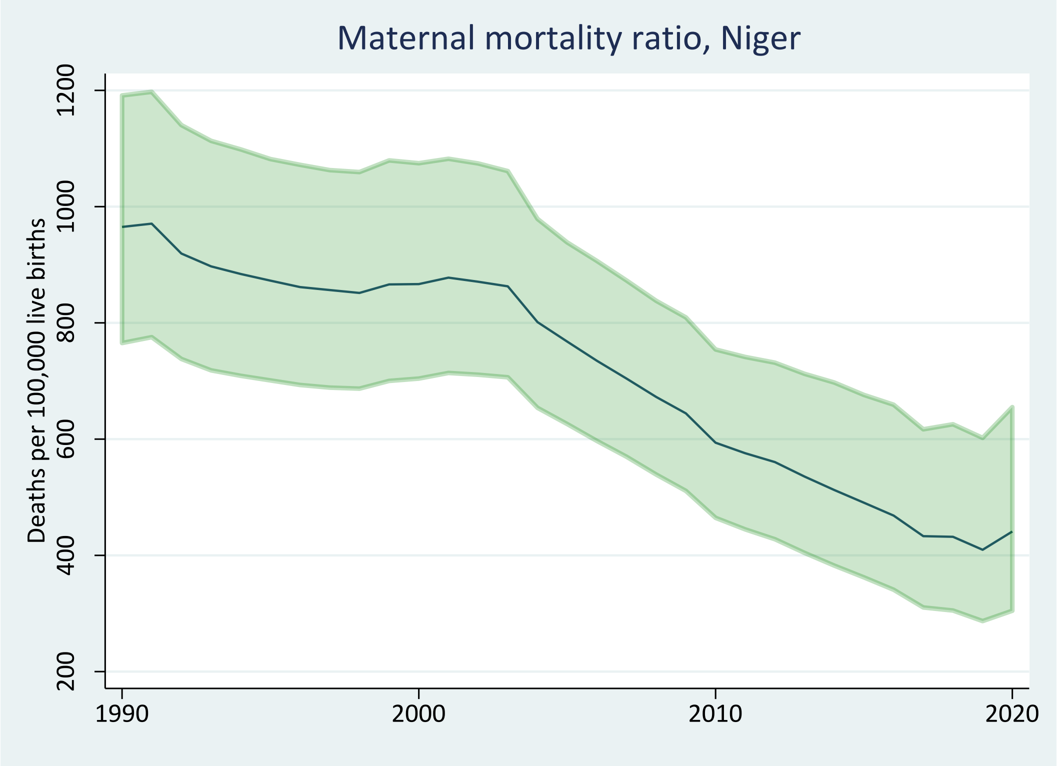

According to the UN-MMEIG estimates, MMR declined by 49% between 2000 and 2020, going from 867 to 441 deaths per 100,000 live births, corresponding to an ARR of 3.4%.

Appendix Figure 4: Proportion of facility-based births by facility type , DHS 1998 and 2021

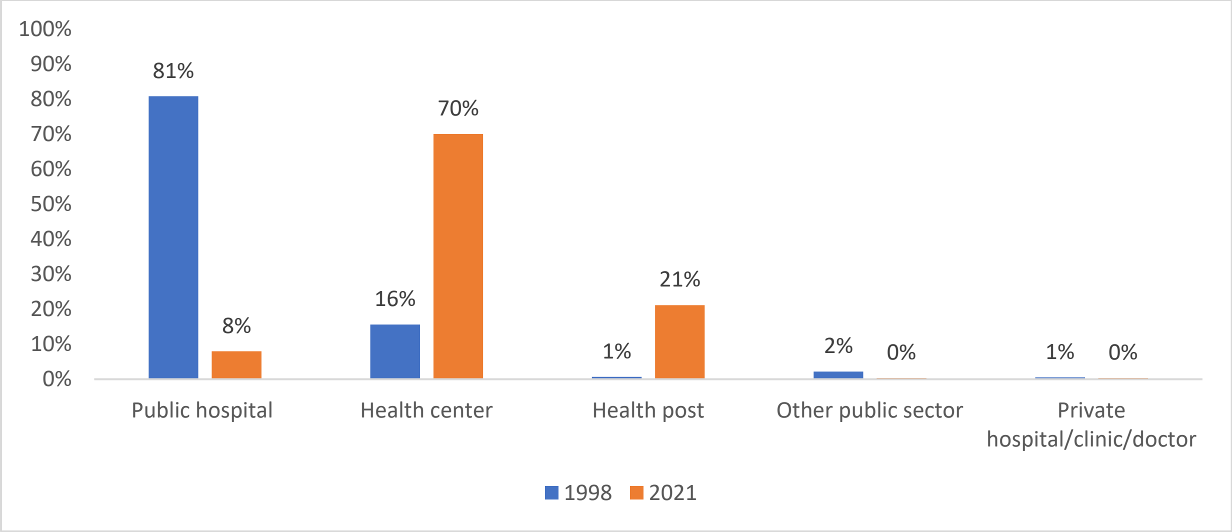

Appendix Figure 5: Trends in coverage equity in key maternal and newborn health (MNH) indicators by household wealth , 1998 - 2021

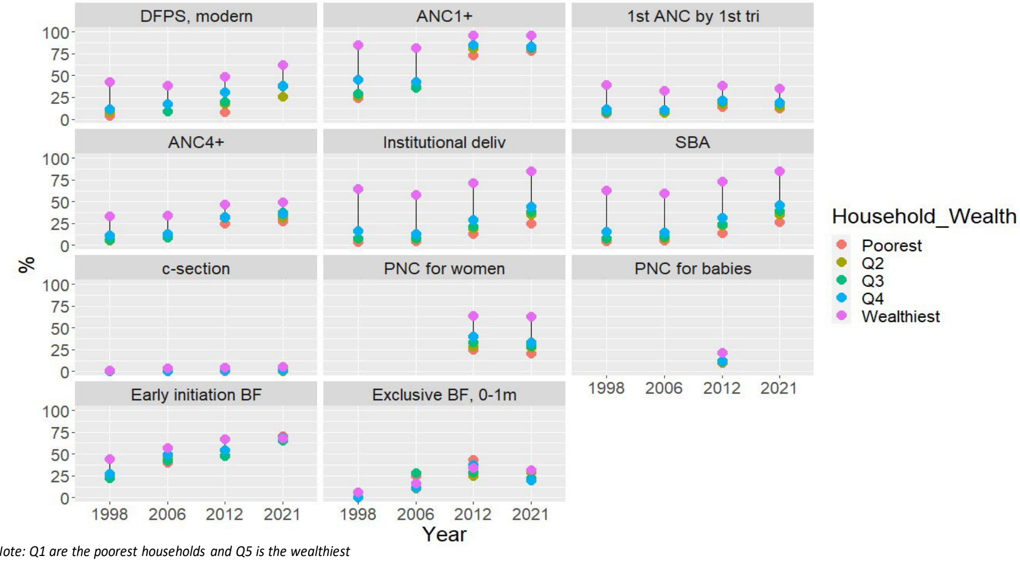

Note: Q1 are the poorest households and Q5 is the wealthiest

Abbreviations: **mDFPS**: Demand for family planning satisfied by modern contraceptive methods (modern methods include pills, condoms (male and female), intrauterine device, sterilization (male and female), injectables, implant, diaphragm, spermicidal agents, patch and emergency contraception); **ANC1+**: One or more visits of antenatal care; **ANC4+**: Four or more visits of antenatal care; **ideliv**: Birth occurred at a health institution/health facility; **PNC-mom**: women received a postnatal check-up within two days post-delivery; **SBA**: skilled birth attendant; **csection**: cesarean section; **EBF**: Infants less than one month of age received only breastmilk in the previous 24 hours; **TT**: baby was born protected from tetanus toxoid infection; **Early BF**: Baby was breastfed in the first hour after delivery; **PNC-baby**: Baby received a postnatal check-up within two days post-delivery

Appendix Figure 6: Trends in coverage equity in key maternal and newborn health (MNH) indicators by mother education , 1998 - 2021

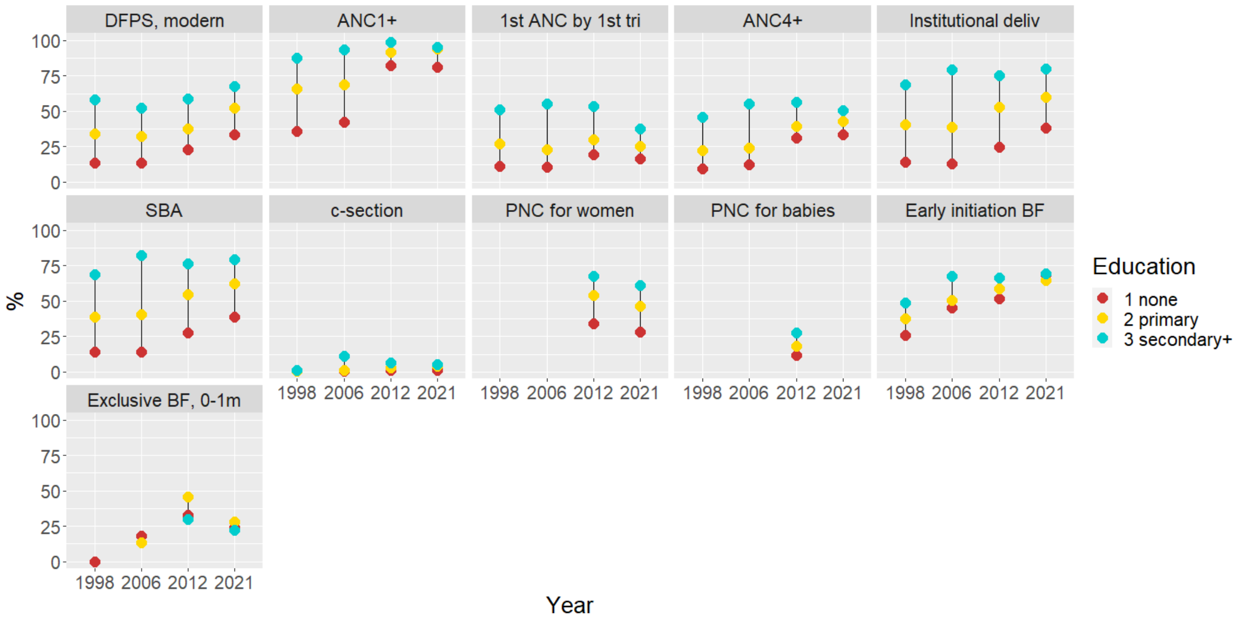

Abbreviations: **mDFPS**: Demand for family planning satisfied by modern contraceptive methods (modern methods include pills, condoms (male and female), intrauterine device, sterilization (male and female), injectables, implant, diaphragm, spermicidal agents, patch and emergency contraception); **ANC1+**: One or more visits of antenatal care; **ANC4+**: Four or more visits of antenatal care; **ideliv**: Birth occurred at a health institution/health facility; **PNC-mom**: women received a postnatal check-up within two days post-delivery; **SBA**: skilled birth attendant; **csection**: cesarean section; **EBF**: Infants less than one month of age received only breastmilk in the previous 24 hours; **TT**: baby was born protected from tetanus toxoid infection; **Early BF**: Baby was breastfed in the first hour after delivery; **PNC-baby**: Baby received a postnatal check-up within two days post-delivery

Appendix Figure 7: Trends in coverage equity in key maternal and newborn health (MNH) indicators by residence area, 1998 - 2021

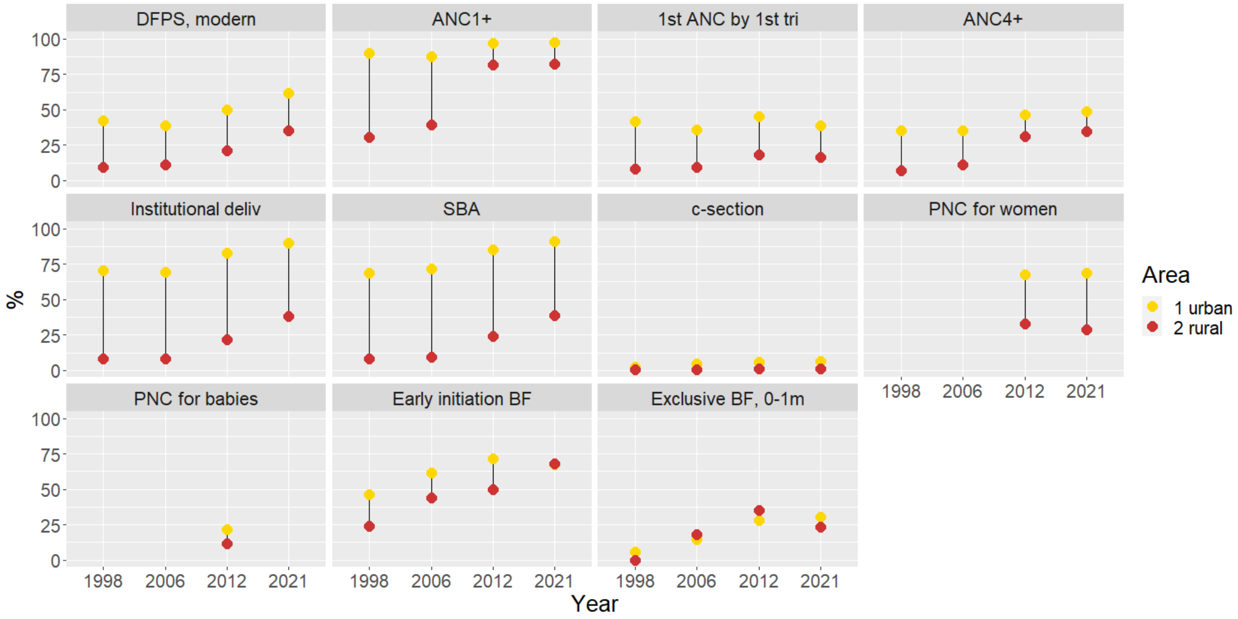

Abbreviations: **mDFPS**: Demand for family planning satisfied by modern contraceptive methods (modern methods include pills, condoms (male and female), intrauterine device, sterilization (male and female), injectables, implant, diaphragm, spermicidal agents, patch and emergency contraception); **ANC1+**: One or more visits of antenatal care; **ANC4+**: Four or more visits of antenatal care; **ideliv**: Birth occurred at a health institution/health facility; **PNC-mom**: women received a postnatal check-up within two days post-delivery; **SBA**: skilled birth attendant; **csection**: cesarean section; **EBF**: Infants less than one month of age received only breastmilk in the previous 24 hours; **TT**: baby was born protected from tetanus toxoid infection; **Early BF**: Baby was breastfed in the first hour after delivery; **PNC-baby**: Baby received a postnatal check-up within two days post-delivery

Appendix Figure 8: Trends in coverage equity in key maternal and newborn health (MNH) indicators by region, 1998, 2021

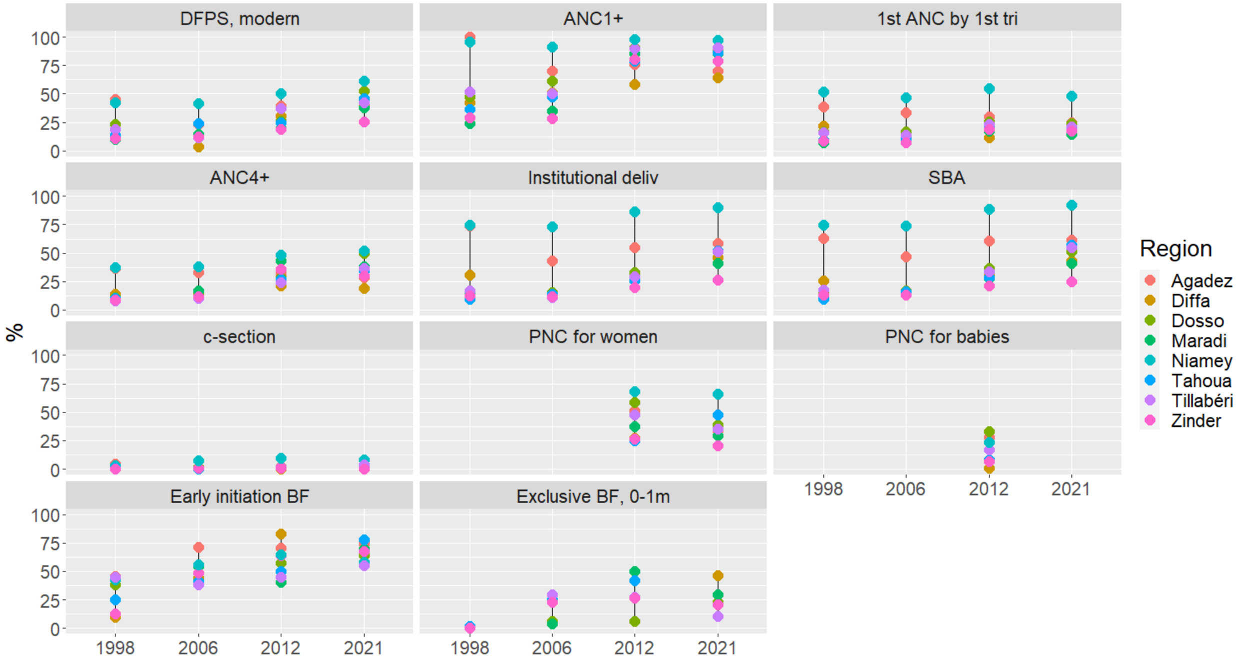

Abbreviations: **mDFPS**: Demand for family planning satisfied by modern contraceptive methods (modern methods include pills, condoms (male and female), intrauterine device, sterilization (male and female), injectables, implant, diaphragm, spermicidal agents, patch and emergency contraception); **ANC1+**: One or more visits of antenatal care; **ANC4+**: Four or more visits of antenatal care; **ideliv**: Birth occurred at a health institution/health facility; **PNC-mom**: women received a postnatal check-up within two days post-delivery; **SBA**: skilled birth attendant; **csection**: cesarean section; **EBF**: Infants less than one month of age received only breastmilk in the previous 24 hours; **TT**: baby was born protected from tetanus toxoid infection; **Early BF**: Baby was breastfed in the first hour after delivery; **PNC-baby**: Baby received a postnatal check-up within two days post-delivery

Appendix Figure 9: Availability of antenatal care (ANC) and delivery services by region, 2019.

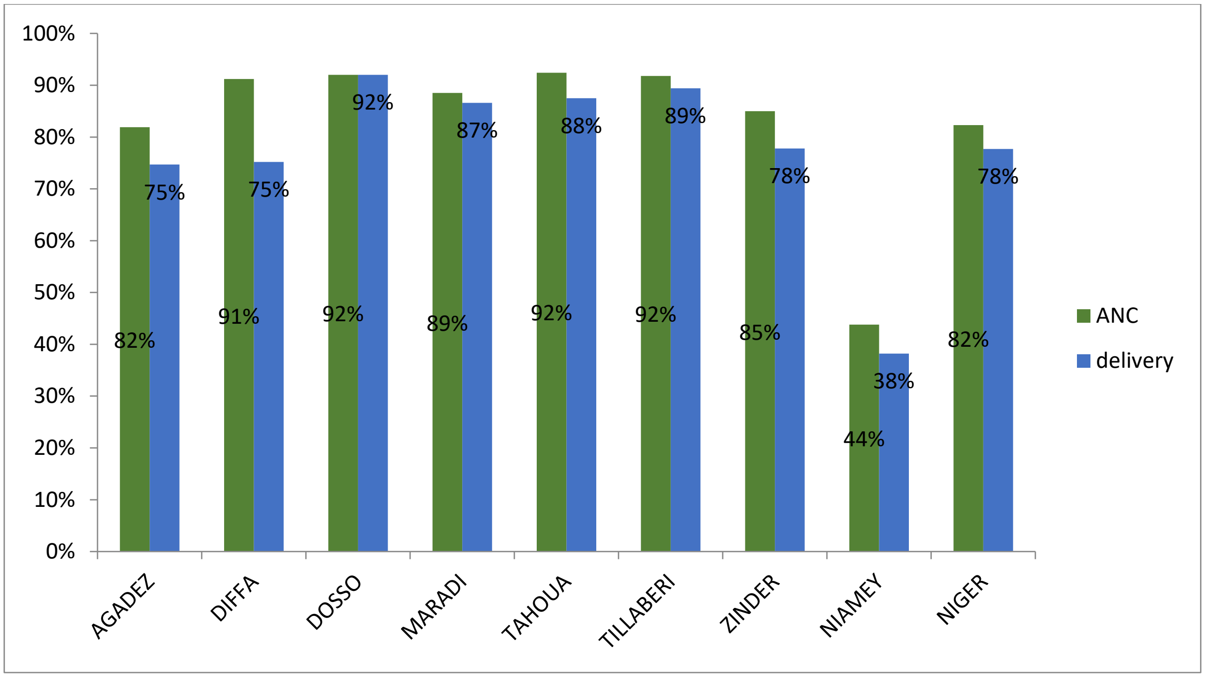

Appendix Figure 10: Trends in antenatal care (ANC) readiness score by region, 2015-2019

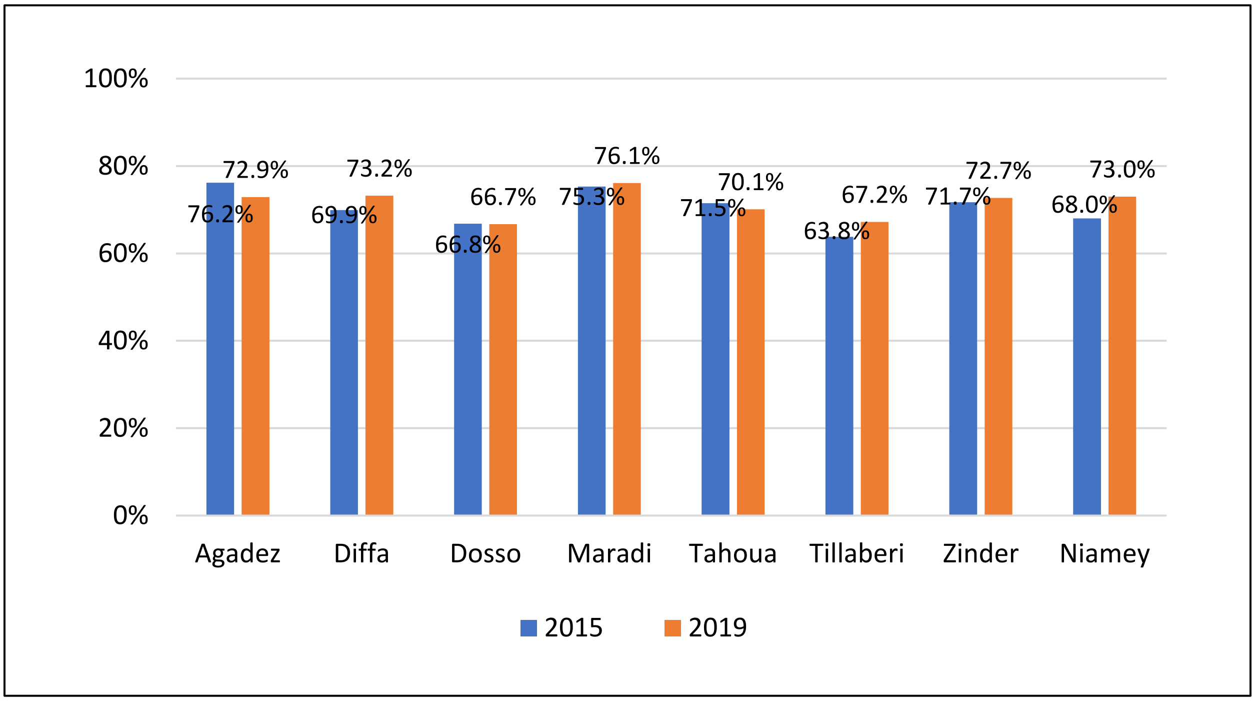

Appendix Figure 11: Basic emergency obstetric and newborn care (BeEmONC) facility readiness for labor & delivery by region, 2015-2019

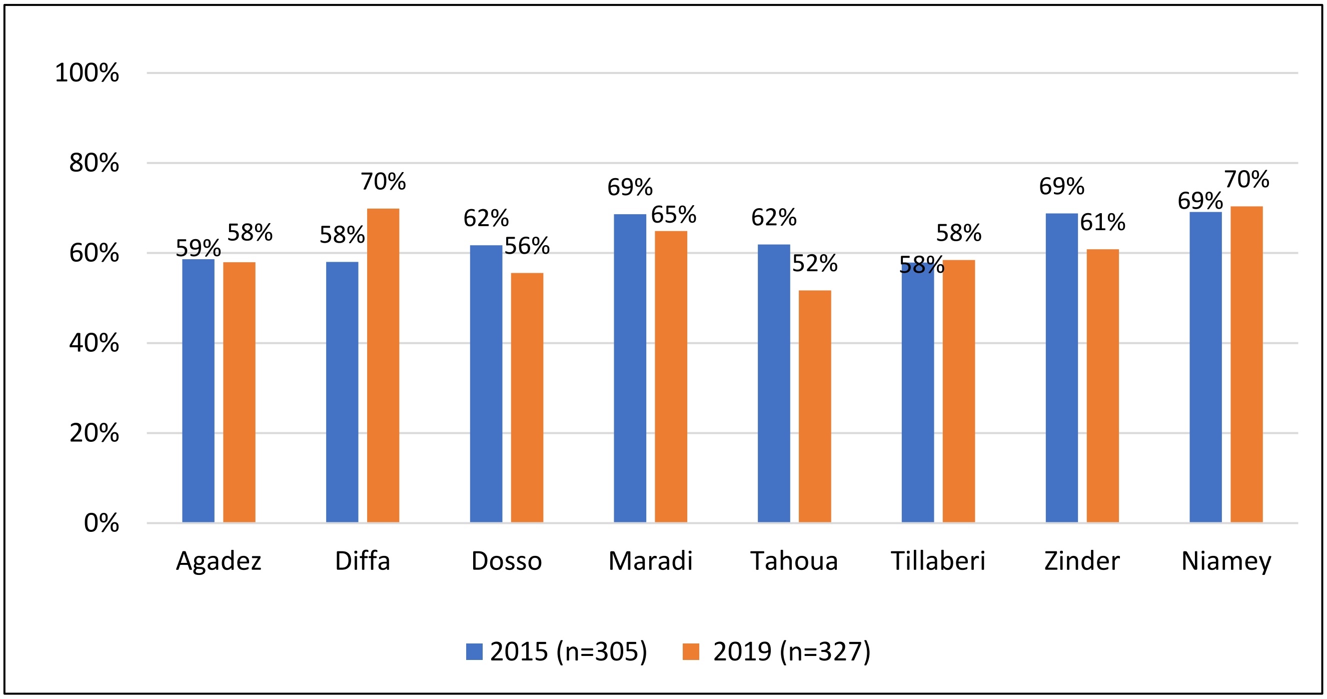

Appendix Figure 12: Antenatal care (ANC) content among women with at least one ANC, 2006-2021

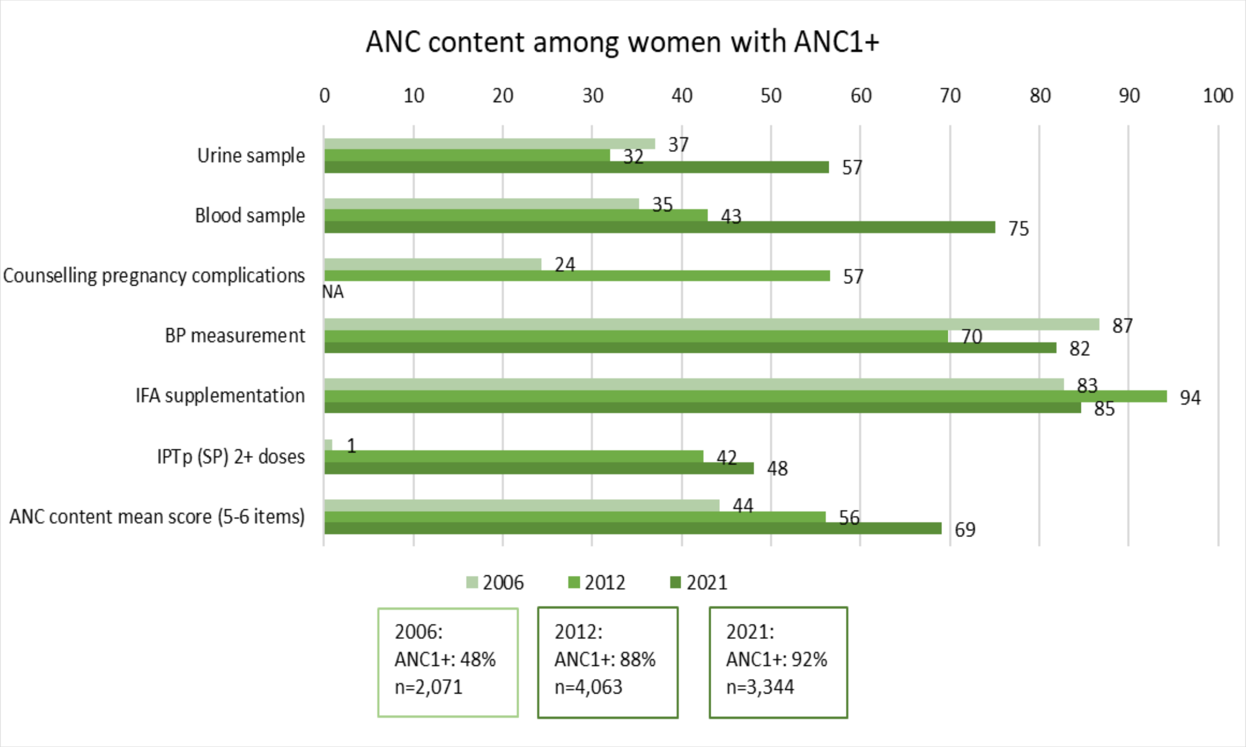

Appendix Figure 13: Readiness adjusted basic emergency obstetric and newborn care (BeEmONC) services by region, 2015

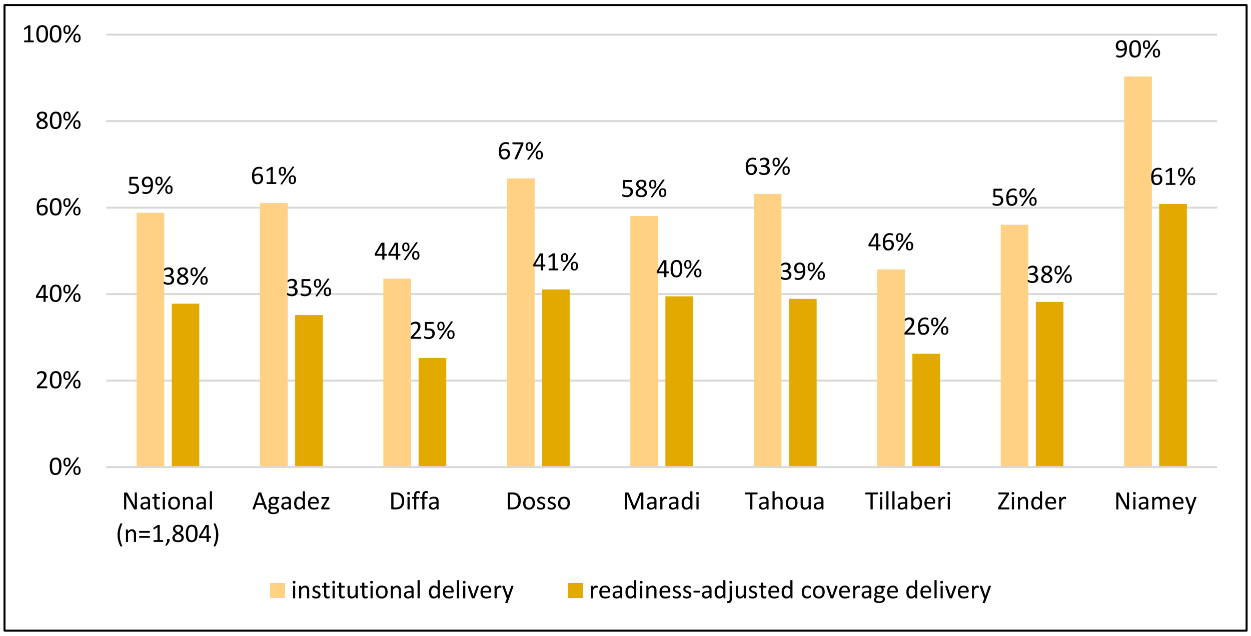

Appendix Figure 14: Readiness adjusted basic emergency obstetric and newborn care (BEmONC) services by region, 2021

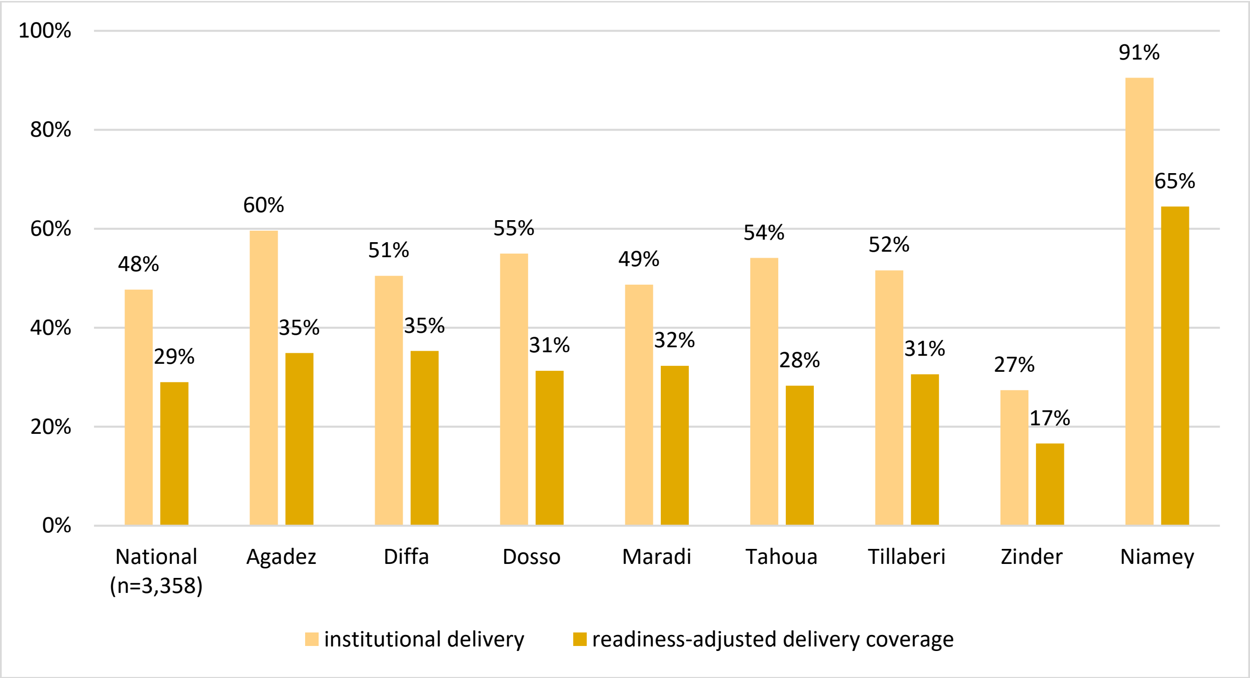

Appendix Figure 15: Contribution of fertility decline (decline in birth rates and changes in birth risk composition) to maternal lives saved and decline in maternal mortality in Niger.

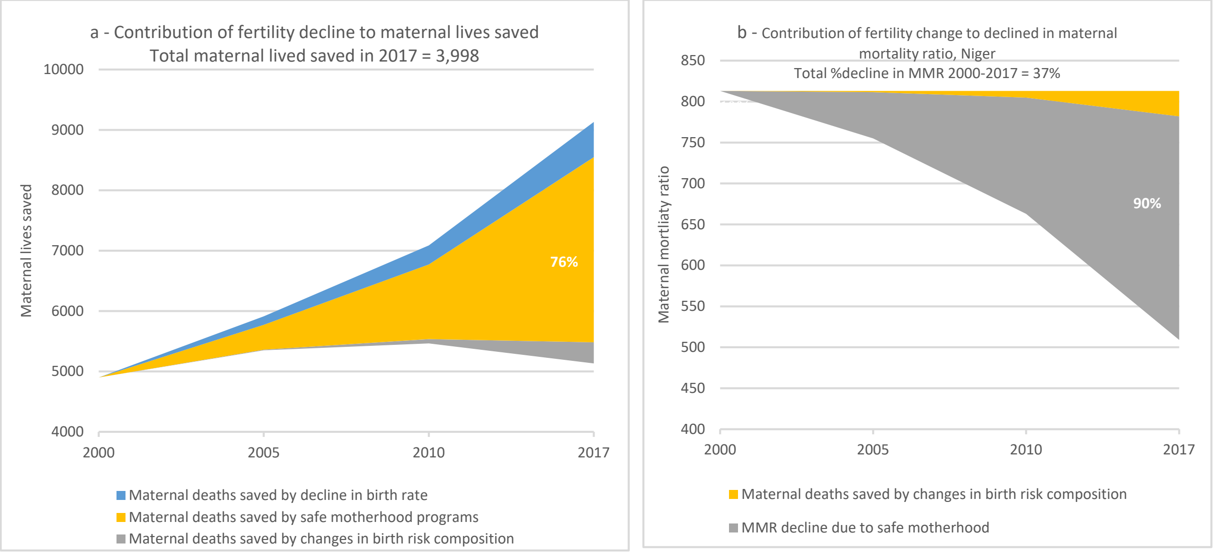

Appendix Figure 16: Contribution of fertility decline (decline in birth rates and changes in birth risk composition) to neonatal lives saved and decline in neonatal mortality in Niger.

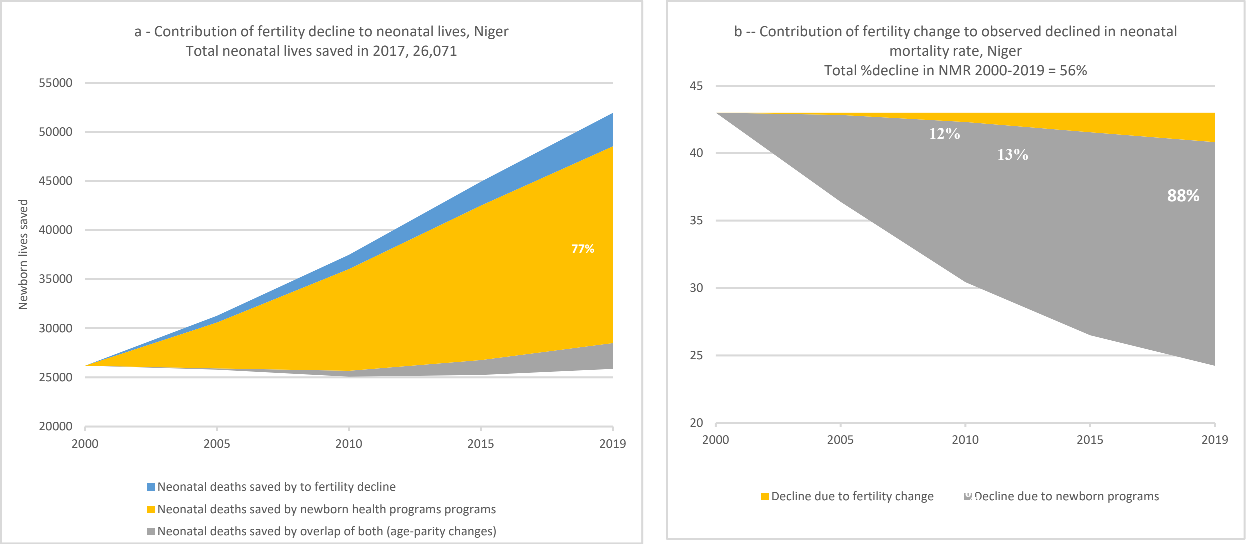

Appendix Figure 17: Niger Neonatal Mortality Rate 2000 to 2021, LiST and IGME estimate

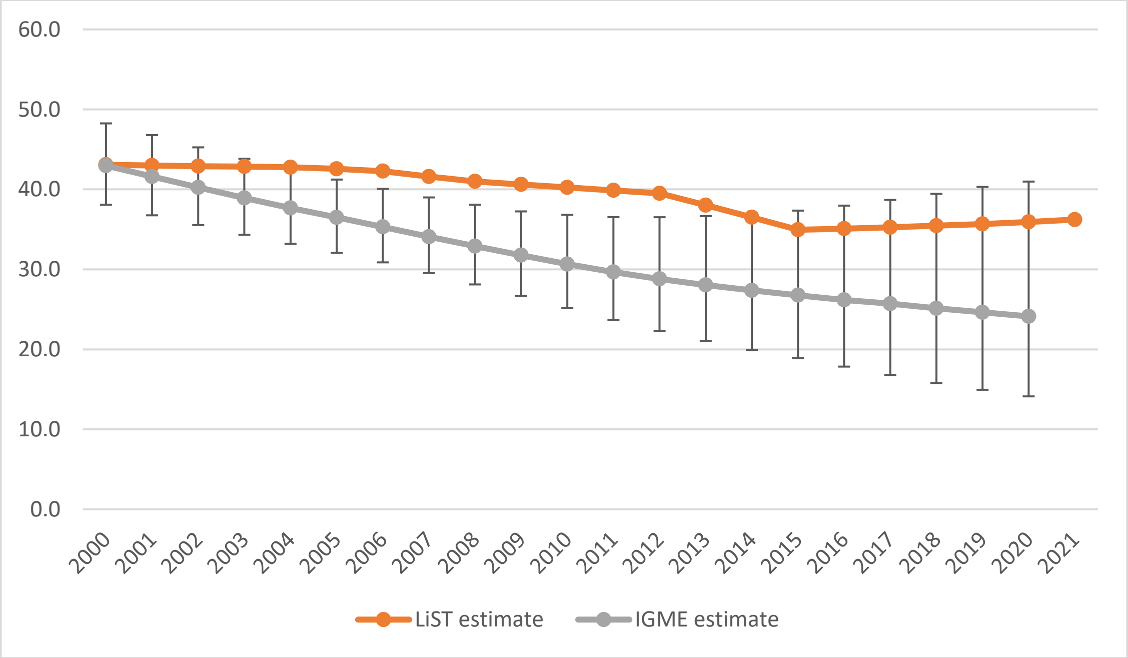

Appendix Figure 18: Niger Maternal Mortality Ratio 2000 to 2021, LiST and UN-MMEIG estimate

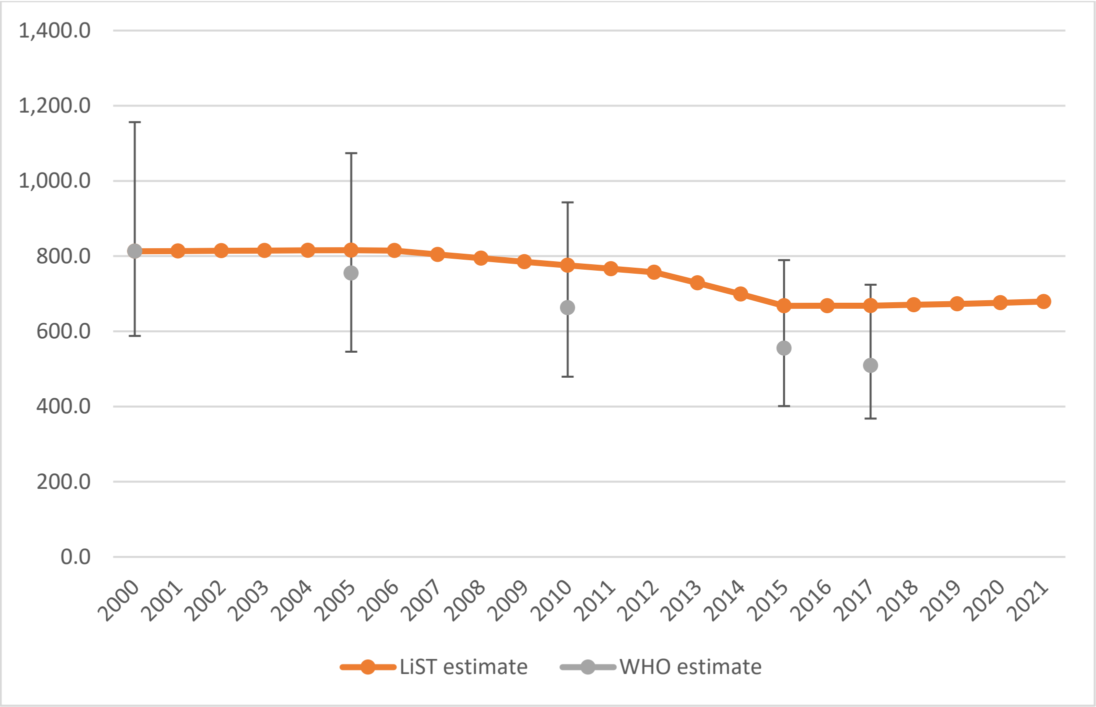

Appendix Figure 19: Health spending in Niger (2000-2018)

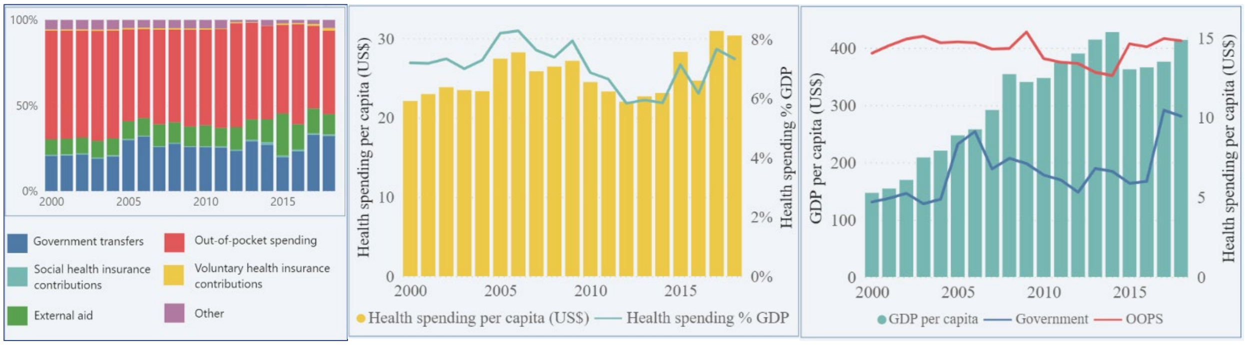

Appendix Figure 20: Number of medical doctors and nurse and midwifery personnel per 10,000 population

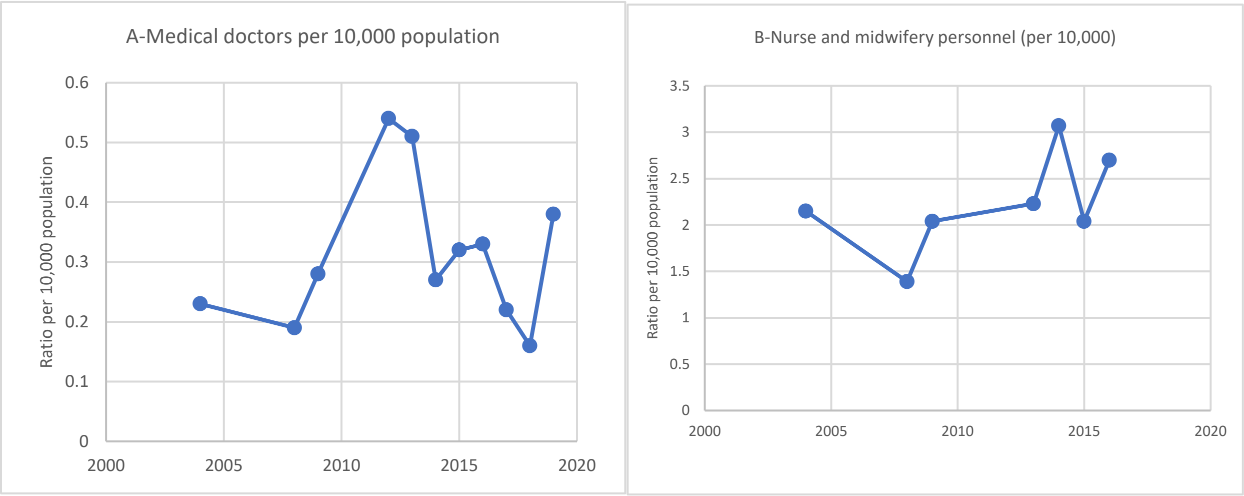

Source: WHO Health Workforce database, https://www.who.int/data/gho/data/themes/topics/health-workforce (accessed May 22, 2022)

Appendix Table

Appendix Table 1: Data sources and use in tables and figures

DATA SOURCES	
**FIGURE**	
Figure 1 A	DHS 2006, 2012, ESM 2010, ENISED 2015, ENAFEME 2021
Figure 1 B	DHS 2006, 2012, ENAFEME 2021
Figure 2	UN-IGME, 2020
Figure 3	DHS 1998, 2006, 2012, ENAFEME 2021
Figure 4	DHS 1998, 2006, 2012, ENAFEME 2021
Figure 5	UN-IGME, 2020
Figure 6	UN-IGME, 2020
Figure 7	PDS (1994-2000 ; 2005-2010 ; 2011-2016 ; 2016-2020) ; PSP (1999-2010) ; SDRP (2008-2012) ; PDES (2012-2017 ; 2017-2021)
**APPENDIX FIGURES**	
Appendix Figure 1	Ministry of health
Appendix Figure 2	UN-IGME, 2020
Appendix Figure 3	UN-IGME, 2020
Appendix Figure 4	UN-MMEIG, 2019
Appendix Figure 5	DHS 1998, ENAFEME 2021
Appendix Figure 6	DHS 1998, 2006, 2012, ENAFEME 2021
Appendix Figure 7	DHS 1998, 2006, 2012, ENAFEME 2021
Appendix Figure 8	DHS 1998, 2006, 2012, ENAFEME 2021
Appendix Figure 9	SARA 2019
Appendix Figure 10	SARA 2015, SARA 2019
Appendix Figure 11	SARA 2015, SARA 2019
Appendix Figure 12	DHS 2006, DSH 2012, ENAFEME 2021
Appendix Figure 13	SONU 2015
Appendix Figure 14	ENAFEME 2021
Appendix Figure 15	IGME
Appendix Figure 16	UN-MMEIG
Appendix Figure 17	WHO Health Workforce databas
**APPENDIX TABLES**	
Appendix Table 1	-
Appendix Table 2	-
Appendix Table 3	SARA 2015, SARA 2019
Appendix Table 4	SARA 2015, SARA 2019

DHS Demographic Health Surveys
ENISED Etude Nationale d'Evaluation d'Indicateurs Socioeconomiques et Demographiques
IGME Interagency Group for Child Mortality Estimation
LiST Lives Saved Tool
MICS Multiple Indicator Cluster Surveys

MMEIG	Maternal Mortality Interagency Group
MMR	Maternal mortality ratio
MNH	Maternal and newborn health
SONU	Soins Obstetricaux et Neonataux d'Urgence
SARA	Service Availability and Readiness Assessment
SPA	Service Provisional Assessment
UN	United Nations
WHO	World Health Organization

Appendix Table 2: Full list of covariates

Level	Variable	Definition	Options	Survey
Distal: Community-level context	Place of residence	Residence in an urban or rural location	Urban - - Rural	DHS
Intermediate: Material circumstances	Household has electricity	Whether the household has electricity or not	No - - Yes	DHS
	Household has landline telephone	Whether the household has a landline telephone or not	No - - Yes	DHS
	Maternal education	Maternal education level with secondary and higher education level merged	None - Primary<br>Secondary or higher	DHS
	Paternal education	Paternal education level with secondary and higher education level merged	None - Primary<br>Secondary or higher	DHS
	Wealth index quintiles	Wealth index is created using 7 dichotomous variables: whether the household owns: (1) a radio, (2) tv, (3) bicycle, and (4) motorbike, and whether the house has an improved material for the (5) floor, (6) wall, and (7) roof. Principal Components Analysis (PCA) was conducted on the pooled sample from both 2000 and 2018. Quintiles were based on 2000 and 2018 surveys separately.	Quintile 1<br>Quintile 2<br>Quintile 3<br>Quintile 4<br>Quintile 5	DHS
	Annual income	PCA was conducted on the pooled sample. Declines were based on 2000 and 2018 separately. Income in each decile was obtained from the International Centre for Equity in Health. Medians determined for 2000 and 2018 separately.	Below median<br>Above median	International Centre for Equity in Health, Pelotas
	Marital status	Current marital status: married, widowed, divorced, no longer living together/separated. Due to small proportions, widowed, divorced, no longer living together/separated were grouped into the "Not married" category.	Married<br>Not married	DHS
	Piped water	Whether the household has piped water as their source of drinking water.	No<br>Yes	DHS

Level	Variable	Definition	Options	Survey
	Open defecation	Whether the household has a toilet facility or not (practicing open defecation).	No<br>Yes	DHS
	Piped water aggregated	Piped water aggregated at the primary sampling unit (PSU) level.<br>Median threshold for year 1998 was applied to both 1998 and 2012	Below median<br>Above median	DHS
	Open defecation aggregated	aggregated at the PSU level.<br>Median threshold for year 1998 was applied to both 1998 and 2012	Below median<br>Above median	DHS
	Improved water	Source of drinking water dichotomized into improved (piped into dwelling, piped into yard/plot, public tab/standpipe, piped to neighbor, tube well or borehole, protected well, protected spring, rainwater, tanker truck, cart with small tank, bottled water), or unimproved (unprotected well, unprotected spring, surface water, other) (1).	No<br>Yes	DHS
	Improved sanitation	Type of sanitation facility dichotomized into improved (flush to piped sewer systems, flush to septic tank, flush to pit latrine, flush to don't know where, ventilated improved pit latrine, pit latrine with slab, composting toilet), or unimproved (flush to somewhere else, open pit latrine, bucket toilet, hanging toilet/latrine, other). No facility/bush/field (open defecation) was not an improved sanitation facility (1).	No<br>Yes	DHS
	Improved sanitation aggregated	Improved sanitation aggregated at the PSU level. Median threshold 1998 was applied to both 1998 and 2012.	Below median<br>Above median	DHS
	Household crowding	Number of household members.	<5 members<br>5+ members	DHS
	People per room	Number of household members divided by number of rooms used for sleeping	Fewer than 3<br>3 or more people	DHS
	Sex of household head	Sex of household head	Male<br>Female	DHS

Level	Variable	Definition	Options	Survey
Intermediate: Behavioural norms and decision-making	Diarrhea care-seeking	Child received treatment or not for diarrhea.	Received treatment<br>Did not receive treatment	DHS
	ARI care-seeking	Child received treatment or not for fever/cough.	Received treatment<br>Did not receive treatment	DHS
	Diarrhea care-seeking aggregated	Aggregated at the sample design strata (SDS) level. Median threshold for year 1998 was applied to both 1998 and 2012.	Below median<br>Above median	DHS
	ARI care-seeking	Aggregated at the sample design strata level. Median threshold for year 1998 was applied to both 1998 and 2012.	Below median<br>Above median	DHS
Intermediate: Health status/need	Risk categories	Four possible risk categories:<br>(1) No risk; (2) unavoidable risk: first birth between 18 & 34 years old, (3) single high risk, any one of: mother's age <18, mother's age >34, birth interval <24 months, birth order >3; (4) multiple high risk: Age <18 years and birth interval <24 months, Age >34 years and birth interval <24 months, Age >34 years and birth order >3, Birth interval <24 months and birth order >3, Age >34 years and birth interval <24 months and birth order >3	No risk<br>Unavoidable risk<br>Single high risk<br>Multiple high risk	DHS
	Delivery by c-section	Delivery by c-section	No<br>Yes	DHS
	Maternal height	Height of woman, with flagged values removed.	minimum to 148<br>148 cm to 152 cm<br>153+ cm	DHS
	Maternal BMI	Height and weight of woman with flagged valued removed. BMI = weight/height2<br>Underweight: BMI<18.5<br>Normal: 18.5<BMI<25	Underweight<br>Normal<br>Overweight and obese	DHS

Level	Variable	Definition	Options	Survey
		Overweight: 25<BMI<30<br>Obese: BMI>30		
	Low birthweight of baby	Size of child at birth	Very large<br>Larger than average<br>Average<br>Smaller than average<br>Very small	DHS
	History of giving birth to twins	Whether the mother had a history of multiple births	No<br>Yes	DHS
	Sex of child	Sex of child	Male<br>Female	DHS
	Child age (months)	Child age in months	No<br>Yes	DHS
	Unwanted pregnancy	Wanted pregnancy when became pregnant: then, later, no more. A pregnancy was unwanted when the woman either wanted the pregnancy later or did not want any more pregnancies.	No<br>Yes	DHS
	Diarrhea prevalence	Child had diarrhea in the past 2 weeks.	No<br>Yes	DHS
	ARI prevalence	Child had cough, short rapid breaths, or problems in the chest or blocked or running nose in the past 2 weeks.	No<br>Yes	DHS
	Fever prevalence	Child had fever in the past 2 weeks.	No<br>Yes	DHS
	Diarrhea prevalence aggregated	Aggregated at the sample design strata (SDS) level. Median threshold for year 1998 was applied to both 1998 and 2012.	Below median<br>Above median	DHS

Level	Variable	Definition	Options	Survey
	ARI prevalence aggregated	Aggregated at the sample design strata (SDS) level. Median threshold for year 1998 was applied to both 1998 and 2012.	Below median<br>Above median	DHS
	Fever prevalence aggregated	Aggregated at the sample design strata (SDS) level. Median threshold for year 1998 was applied to both 1998 and 2012.	Below median<br>Above median	DHS
Proximal: Intervention coverage	4+ ANC visits	Four or more antenatal care visits	No<br>Yes	DHS
	Composite (ANC, SBA, HFD)	Composite of four or more antenatal care visits, skilled birth attendance, and health facility delivery	0 – 1 – 2 - 3	DHS
	Skilled birth attendance (SBA)	Skilled attendant at birth (assistance from a doctor or nurse/midwife/paramedic)	No<br>Yes	DHS
	Modern contraceptive method	Current use of contraceptive by type: no method, folkloric method, traditional method, or modern method. Variable was dichotomized to modern method or the rest.	No<br>Yes	DHS
	Health facility delivery (HFD)	Place of delivery: public or private health facility (1).	No<br>Yes	DHS
	EIBF	Early introduction of breastfeeding; breastfed within 1 hour of birth.	Within 1 hr<br>>1 hr	DHS

Appendix Table 3: Antenatal care, BeMONC and CeMONC readiness items by domain.

Antenatal Care	
Domain	Items
Equipment	Blood pressure apparatus<br>Stethoscope<br>Latex gloves<br>Single use syringe<br>Soap and water or alcohol-based rub

	Environmental disinfectant
Diagnostics	Hemoglobin testing<br>Urine dipstick protein<br>Urine dipstick glucose<br>Syphilis testing<br>HIV testing
Medicines & commodities	Iron tablets<br>Folic acid tablets<br>Tetanus toxoid vaccine<br>Insecticide-treated bed nets<br>Intermittent prevention and treatment for malaria during pregnancy (Sulfadoxine/pyrimethamine)
Basic amenities	Improved water source<br>Improved sanitation facilities<br>Room with auditory and visual privacy
Human resources & guidelines	Trained staff in ANC in previous 2 years<br>ANC guidelines<br>ANC job aids
Basic Emergency Obstetric and Newborn Care (BEmONC)	
Equipment	Latex gloves<br>Examination light<br>Delivery bed<br>Delivery kit<br>Emergency transport<br>Partograph<br>Manual vacuum<br>Vacuum aspirator<br>Sterilization material<br>Newborn bag & mask
Medicines and commodities	Skin disinfectant<br>Injectable antibiotic<br>Intravenous solution with perfusion<br>Injectable uterotonic<br>Injectable magnesium sulphate<br>Antibiotic eye ointment
Human Resources & guidelines	Guidelines for essential newborn care<br>Guidelines for essential childbirth care<br>Staff trained in essential childbirth care in previous 2 years<br>Staff trained in newborn resuscitation in previous 2 years
Comprehensive Emergency Obstetric and Newborn Care (CEmONC)	
Equipment	Oxygen

	Resuscitation table with heat source<br>Incubator<br>Spinal needle
Medicines and commodities	Safe blood<br>Injectable atropine<br>Suxamethonium bromide powder<br>Injectable epinephrine<br>Halothane inhalation<br>Injectable ketamine<br>Anesthesia<br>Thiopental powder<br>Blood group serology testing<br>Blood stock
Human Resources & guidelines	CEmONC guidelines<br>Staff trained in CEmONC in previous 2 years<br>Staff trained in anesthesia in previous 2 years<br>Staff trained in surgery in previous 2 years<br>Staff available for C-section 24 hours

Appendix Table 4. ANC, BEmONC and CEmONC readiness by facility type in 2015 and 2019.

**Facility type & managing authority**	**ANC readiness (%)**		**BEmONC readiness (%)**		**CEmONC* readiness (%)**	
	2015 (n=304)	2019 (n=316)	2015 (n=305)	2019 (n=327)	2015 (n=41)	2019 (n=52)
**Public Hospital/maternity/maternal & child center**	75.5	84.5	82.8	80.4	78.6	75.9
**Private Hospital/polyclinic**	71.7	68.5	73.9+	57.3+	69.1+	67.7+
**Public Integrated Health Centers (IHC)**	69.8	71.1	61.8	58.7	-	-
**Private cabinet**	71.5	72.7				
**National level mean**	69.9	71.1	63.0	59.7	75.4	74.8

Antenatal care (ANC)

Basic Emergency Obstetric and Newborn Care (BEmONC)

Comprehensive Emergency Obstetric and Newborn Care (CEmONC)

*CEmONC readiness restricted to facilities offering blood transfusion and C-section services

+ Includes Private cabinets
